# Hypersensitive pressure sensors inspired by scorpion mechanosensory mechanisms for near-body flow detection in intelligent robots

**DOI:** 10.1126/sciadv.ady5008

**Published:** 2025-08-20

**Authors:** Pinkun Wang, Changchao Zhang, Bo Li, Xiancun Meng, Yuechun Ding, Junqiu Zhang, Shichao Niu, Zhiwu Han, Liwei Lin, Luquan Ren

**Affiliations:** ^1^ Key Laboratory of Bionic Engineering (Ministry of Education), Jilin University, Changchun, Jilin, 130022, China.; ^2^ Liaoning Academy of Materials, Liaoning, Shenyang, 110167, China.; ^3^ Department of Mechanical Engineering and Berkeley Sensor and Actuator Center, University of California at Berkeley, Berkeley, CA 94720-1740, USA.

## Abstract

Sensitivity enhancement for pressure sensors over a broad linear range can improve sensing performance for a wide range of applications such as health monitoring and artificial intelligence. Here, inspired by the high-precision mechanosensory mechanism of the scorpion, a bioinspired piezoresistive pressure sensor (BPPS) is reported for the synergistic enhancement of sensitivity and linearity at 65.56 millivolts per volt per kilopascal and 0.99934, respectively, in a pressure range from 0 to 500 kilopascals. The BPPS can distinguish laminar, transitional, and turbulent flows as well as identify approaching objects of different shapes with an accuracy exceeding 85.42% by integrating a wavelet transform algorithm and the ResNet18 deep learning network. As a proof of concept, BPPS has been engineered in a hexapod robot to enable near-body flow field sensing for active collision avoidance. This work underscores the potential to leverage key design concepts inspired by living insects for improved sensing performance and offers structural insights for other high-precision sensors.

## Teaser

Scorpion-inspired high precision pressure sensors can distinguish near-field flows for active collision avoidance in robotics.

## INTRODUCTION

Pressure sensors can transduce pressure stimuli into electric signals with various promising applications in health monitoring (*1*–*5*), intelligent robotics (*6*–*11*), and human-machine interaction (*4*, *6*, *12*, *13*). Silicon-based piezoresistive pressure sensors are well-studied and widely used commercial products owing to their high durability, low energy consumption, and batch fabrication capability (*14*–*16*). Although substantial advancements have been achieved in developing piezoresistive pressure sensors with high performance, the trade-off between sensitivity and linear range remains a huge challenge and affects sensing precision (*17*–*19*). Specifically, the sensitivity of piezoresistive pressure sensors is directly determined by the stress in the piezoresistors, and conventional designs use special beam or island structures to amplify deformations, but they also exacerbate the balloon effect (i.e., increased axial stress in the membrane), which can notably reduce the linearity (*20*). Furthermore, large deformations can induce grain boundary friction, dislocation, and the propagation of defects, such that a substantial amount of energy is dissipated in the inelastic potential, which results in a decrease in linearity (*21*, *22*).

**Fig. 1.**
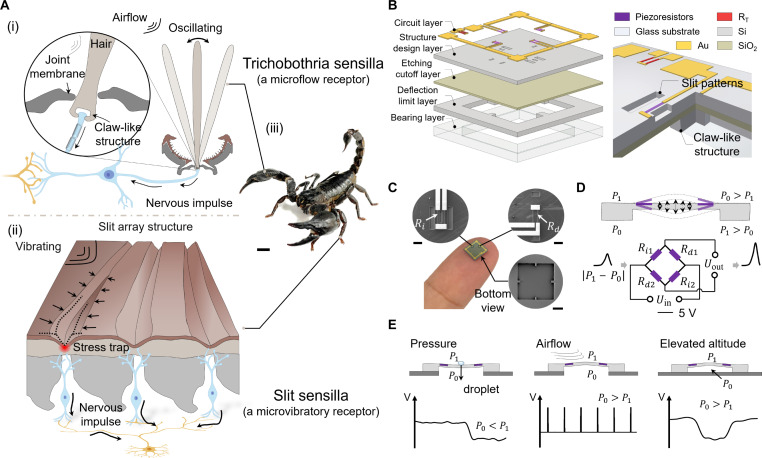
High-precision pressure sensor inspired by the mechanical sensilla of scorpions. (**A**) Somatosensory system of the scorpions. (i) Trichobothria sensilla. (ii) Slit sensilla. (iii) *H. petersii* scorpion. Scale bar, 1 cm. (**B**) Structures of the BPPS and (**C**) digital image of BPPS and SEM images of bioinspired stress traps (frontside) and flexure suppression units (backside). Scale bars, 100 μm; 100 μm; and 1 mm. Resistors in these figures: *R*_*x* (*x*=*i*,*d*,*T*)_. *i*, increase; *d*, decrease; *T*, temperature. (**D**) Sensing principle. When external stimuli induce a pressure differential across the sensing membrane, the BPPS will output a corresponding voltage. (**E**) BPPS exhibits a versatile detection capability across multiple physical quantities, such as pressure, airflow, and elevated altitude.

In nature, organisms have evolved excellent perceptual abilities with exceptional sensing capabilities to accommodate complicated and harsh living environments, which provides inspiration for the development of artificial sensing systems (*13*, *23*–*25*). Scorpions, with their faded vision, have evolved a high-precision mechanosensory system composed of trichobothria sensilla to perceive changes in airflow and slit sensilla to detect vibrations from the ground (Fig. 1A and movie S1). The scorpion trichobothria sensillum is characterized by long, slender hair shafts (figs. S1 and S5). The root of the trichobothria sensillum exhibits a claw-like structure formed because of localized hyperplasia, with the claw tips directly contacting the receptors at the synaptic terminals of the nerve endings (*26*). In contrast to the conventional hair-shaped mechanoreceptors, the claw-like root structure effectively suppresses the deflection displacement of the sensory membrane, thereby enabling scorpions to perceive pressure signals in a highly linear manner (*27*, *28*). On the other hand, the slit sensillum is composed of 12 slits radiating outward in the exoskeletal plane, along with mechanosensory neurons positioned beneath the slit tips (figs. S2 to S4). When vibrational signals stimulate the slit sensilla, the slits effectively facilitate the convergence of dispersed mechanical energy, similar to how scorpions construct a stress trap (ST) to capture stress. The corresponding neuron converts the mechanical energy into membrane potential (*29*–*31*). Furthermore, the high overlap between the stress concentration region and the receptive field of neurons, known as the “spatial principle,” also notably enhances the scorpion’s sensitivity to perceive vibrations in the surrounding environment (*32*). Therefore, the claw-like structure and slit structure of the scorpion enable it to achieve remarkable precision in perception, aiding its proficiency in capturing prey and evading predators.

In general, sensors are stimulated by external stimuli, which excite the sensitive elements to generate electrical signals. However, the energy required to activate the sensitive elements is often insufficient, and internal friction in the sensitive elements reduces the elastic potential energy, resulting in reduced sensitivity and linearity. Drawing inspirations from the somatosensory system of the scorpion (*Heterometrus petersii*), stress traps around the sensitive elements are used to enhance the overall energy influx for improved sensitivity. On the other hand, claw-like structure inspires the design of flexure suppression units to reduce the nonlinear internal friction induced by large deformation for increased linearity. Together, a bioinspired piezoresistive pressure sensor (BPPS) with vertically oriented flexure suppression units and horizontally distributed stress traps has been developed with a Wheatstone bridge configuration (Fig. 1B) to synergistically enhance its sensitivity and linearity. At the top silicon layer of the silicon-on-insulator (SOI) chip, horizontally distributed stress traps are arranged around the four piezoresistors to boost their sensitivity, while vertically oriented flexure suppression units are fabricated on the bottom silicon substrate to improve the linearity by suppressing large displacements. Digital images of BPPS and scanning electron microscopy (SEM) images of bioinspired stress traps (frontside) and flexure suppression units (backside) are shown in Fig. 1C. A temperature resistor (*R*_T_) is also placed at the nondeforming region to mitigate the temperature effect on the piezoresistive sensor, and Fig. 1D shows the Wheatstone bridge setup. This bioinspired structural design endows the BPPS with superior sensing performance, including high sensitivity (65.56 mV/V per kPa) and linearity (with a coefficient of determination of 0.99934) for a pressure range from 0 to 500 kPa, fast response time (10 ms), fast recovery time (4 ms), and remarkable durability (more than 20,000 cycles). Accordingly, the BPPS is shown to precisely detect multiple physical quantities (such as pressure, airflow, and altitude), contingent upon variations in pressure differentials across the membrane surface (Fig. 1E). In addition, the BPPS is able to identify the flow regime between laminar, transitional, and turbulent flows. Implementing a machine learning algorithm to analyze the data acquired by the BPPS enables 85.42% accuracy in identifying five approaching objects with different shapes. As a proof of concept, BPPSs are applied in hexapod walking robots to mimic the scorpion’s hunting and predator evasions. These advancements hold substantial potential for applications such as flow perception for intelligent robots.

## RESULTS

### Scorpion mechanosensory mechanisms and simulation optimization

**Fig. 2.**
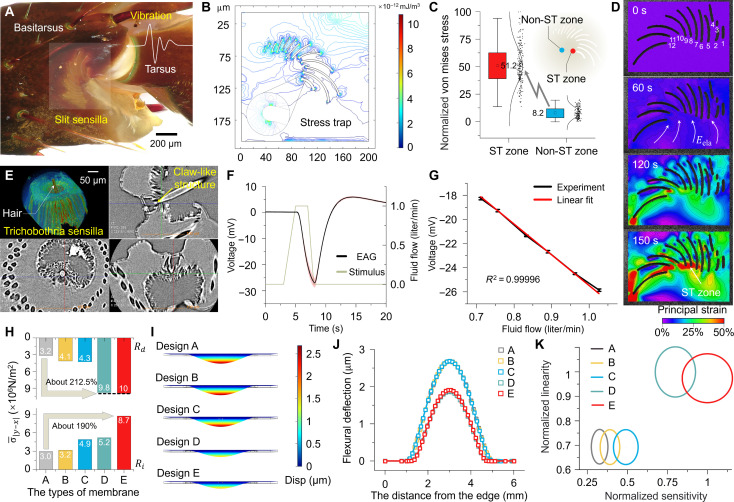
Revelation of scorpion mechanosensory mechanisms and simulation optimization. (**A**) A magnified view of slit sensillum. The white areas in the diagram are the slit sensillum sites. (**B**) Stress traps distributed at the tips of slit sensillum. (**C**) Normalized von mises stress comparison between stress trap area and other areas. (**D**) DIC results. The deformation is mapped with the Hencky strain. (**E**) A 3D μ-CT reconstructed trichobothria sensilla structure. The claw-like structure is the end of the trichobothria, directly contacting the receptors at the synaptic terminals of the nerve endings. (**F**) Electrophysiological response of a trichobothria sensilla under an airflow of 1.026 liter/min. (**G**) Dependence of EAG amplitudes on the airflow (0.700 to 1.050 liter/min), along with its linear fitting curve. (**H**) The average stress of *R_i_* and *R_d_* when they are positioned optimally in designs A to E. (**I**) The finite element strain cloud map of the sensing membrane under an imposed constant pressure of 1 kPa. (**J**) The flexural deflections of the membrane with *y* set to 3000 mm. The maximum deflection displacement in design E is notably reduced. (**K**) Visualization of sensitivity and linearity in designs A to E.

The somatosensory system of the scorpion comprises slit sensilla and trichobothria sensilla. As shown in Fig. 2A, the slit sensilla, located at the distal end of the basitarsus, detect microvibrations from the substrate transmitted through the tarsus. The unique structural morphology of these slits piqued our interest to use COMSOL for proportionate modeling and steady-state simulation using the solid mechanics module. The results of the elastic strain energy density are depicted in Fig. 2B, where the strain energy at the slit tip zone is observed to be six times greater than that in other regions, a phenomenon we refer to as a “stress trap” (Fig. 2C). To validate the effectiveness of our simulation, we conducted compression tests on a three-dimensional (3D) printed prototype of the model and used digital image correlation (DIC) technology to analyze the stress distribution throughout the process (Fig. 2D). It was observed that starting from 60 s, the stress began to concentrate toward the slit tip, with the stress trap region becoming evident at 150 s. This strategy of constructing stress traps to modulate the stress distribution around sensitive elements has inspired our design approach for enhancing sensitivity.

The spatial structure of trichobothria sensillum was obtained through 3D micro-computed tomography (μ-CT) scanning in Fig. 2E. The trichobothrial shaft extends from a honeycomb-like trichobothrial socket, with its base evolving into a specialized claw-like structure that connects the dendrites of sensory nerve endings. The presence of this claw-like structure minimizes the deformation required to elicit action potentials in the sensory nerve endings in response to external perturbations and reduces unnecessary nonlinear effects. Electrophysiological test for electroantennogram (EAG) signals from whole scorpion pedipalps without dissection for the trichobothria sensilla (in situ) under different levels of airflow stimuli at 23.5°C shows good linear response. A Faraday cage was used to shield the external electromagnetic waves, and several filtering algorithms were used to eliminate noises originating from the scorpion itself (above 1 kHz) for signals within the 0.1-Hz to 1-kHz range. The range of airflow velocities (0.700 to 1.050 liters/min) encountered in the natural habitat of the *H. petersii* scorpion was covered during the testing to better capture the electrophysiological signals generated in its natural state (fig. S6). Figure 2F demonstrates the EAG of the scorpion under airflow stimulation at 1.026 liters/min, with a maximum amplitude of ~26 mV. The red shaded area indicates the 95% confidence interval derived from 10 repeated experiments. The study of the dependence on the EAG of scorpion trichobothria sensilla under different airflow stimulation shows that the trichobothria sensilla exhibit an exceptional linearity of 0.99996 (Fig. 2G). This indicates that the unique claw-like structure notably helps modulate the nonlinear energy dissipation within the sensilla, distinguishing it from other hair-type sensory receptors.

To apply scorpion mechanosensory mechanisms to sensor design, we conducted a series of simulation studies. These simulations aimed to emulate the unique structural and functional characteristics of scorpion sensory sensilla for enhancing sensor performance. The synergistic improvement in sensor sensitivity and linearity is directly related to the concentration of stress distribution around piezoresistors and the reduction in the flexural deflection of the membrane. The higher the stress concentration, the greater the change in piezoresistor resistance under external stimulation. On the other hand, the greater the flexural deflection, the greater the relative slip in the lattice structures of silicon and the more microcosmic friction heat to deviate from linear responses. Briefly, the higher the concentration of stress, the higher the sensor’s sensitivity. The shorter the flexural deflection, the higher the linearity (*33*, *34*).

Finite element analysis (FEA) models for both horizontally distributed stress traps and vertically oriented flexure suppression units were developed to investigate their effects on the stress concentration of the four piezoresistors. The stress state analyses around the stress traps at resistors were conducted, followed by geometric morphology optimizations (figs. S11 to S16). The optimization was conducted for the number, length, and arrangement pattern of the stress traps built around piezoresistors, as well as the length of the flexure suppression units. To ascertain the sensing benefits of the bioinspired architecture, comparisons were conducted between the performance of its geometry and that of four other membrane structures, all with the same total size and the same total material to ensure a fair comparison. More specifically, we considered design A, with no stress trap and no flexure suppression unit; design B, with single slit elements widely found in modern engineering applications; design C, with only stress traps; design D, with only the flexure suppression unit; and design E, inspired by the scorpion’s specialized somatosensory structures with both stress traps and flexure suppression units (fig. S18). The optimal configuration of the sensitive units for designs A to E has been studied to achieve maximum sensing precision (figs. S19 to S28). The setting parameters of the FEA models and geometrical dimensions are described in fig. S29 and tables S1 and S2. Four piezoresistors are placed parallel to the four sides of the square membrane to adopt different slit structures for two sets of piezoresistors (*R_i_* and *R_d_*) with different strain states, rather than just considering the degree of stress concentration (notes S1 to S4 and figs. S10 and S17). Hence, for *R_i_*, we opted for the slit 9 type with the highest stress confinement efficacy (fig. S15). As for *R_d_*, we selected the slit 2 type, which exhibits excellent stress confinement capability and offers the advantage of facilitating the layout of piezoresistors (fig. S16).

Figure S27 illustrates the difference σ*_|y−x|_* of the second Piola-Kirchhoff stress component σ*_x_* and σ*_y_* on the stress cutoff line (*y* = 3000 μm) of five different types of square membrane surfaces at an imposed constant pressure *P* = 1 kPa. It was evident that the membrane experiences substantial stress within the range of *x* = 1100 to 1500 μm. Thus, this area was designated as the layout zone for *R_i_*. In the research on the arrangement of piezoresistors, two indicators were considered comprehensively: the degree of stress concentration and the uniformity of stress distribution. As shown in figs. S23 and S24, *y* = 1030 μm has been chosen as the *R_d_* distribution line for designs A to C and *y* = 1310 μm as the *R_d_* distribution line for designs D and E. By graphically representing the stress difference values σ*_|y−x|_* of various designs, the optimal setting for *R_d_* can be visually demonstrated on the respective stress cutoff line. Design E exhibits intense stress concentration and highly uniform stress distribution in the *x* = 2.9 to 3.1 mm range (fig. S28). Figure 2H presents the average stress of *R_i_* and *R_d_* in the optimal placement region for the five designs. In the current study of the two groups of piezoresistors, the biomimetic design E exhibits the highest degree of stress concentration and the most uniform stress distribution for high sensitivity.

The investigation of linearity on five types of membrane-structured sensors focuses on the deflection displacement of the membrane. Under a pressure of 1 kPa, the finite element strain cloud maps of the five membrane samples and the strain values on the *y* = 3000 μm cross section are displayed in Fig. 2 (I and J). By designing the vertically oriented flexure suppression units, designs D and E exhibit the smallest deflection displacement of 1.85 and 1.91 μm with a reduction of ~31 and 29% compared to other designs with a 2.69-μm deflection. When the upper surface of the silicon film is subjected to pressure, the internal lattice of the film deforms downward to generate reverse stress to balance external excitation. The flexure suppression units in designs D and E contribute to the reaction force to suppress the bending deformation and reduce the loss of linear energy. The flexure suppression units can reduce flexural displacement, but they also induce stress instability such that their size is discussed in detail in figs. S25 and S26. The stress values for the two sets of piezoresistors are averaged and used as a reference indicator for the sensitivity of the corresponding membrane. The maximum flexural deflection of the membrane is taken as the reference index for nonlinearity. These two reference indices are normalized and denoted as Ns and Nl, respectively, and plotted in Fig. 2K. Geometric ellipses are used for visualizing the sensitivity and linearity reference indicators of each design, serving as the center values of the geometric ellipse and the major and minor axis values of the response. The addition of a stress trap (design E) in design D notably enhances the sensitivity of the sensor (20%) while slightly sacrificing linearity (2%). This trade-off is acceptable for high-precision sensors.

### Configuration and characterization of BPPS

**Fig. 3.**
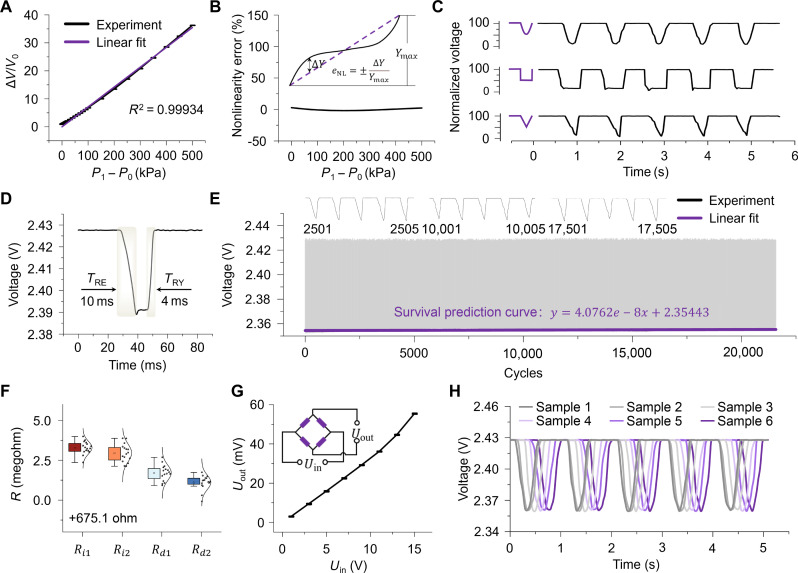
Characterizations of BPPS. (**A**) The response curve of the BPPS under static pressures (0 to 500 kPa), along with its linear fitting curve. (**B**) The nonlinear error between the experimental curve and the linear fitting curve with an intercept of 0. (**C**) The recognition of three different input vibration waveforms (1 Hz) by BPPS implies its capability to identify distinct vibration signals. (**D**) The response speed of BPPS under 1-Hz half square wave impulse. (**E**) Fatigue test results of BPPS under repetitive 1-Hz half triangular wave impulses for more than 20,000 cycles and peak fitting curve for survival prediction. (**F**) The consistency of BPPS resistance values. The range of resistance values among a randomly selected set of 16 BPPSs is less than 0.3‱. (**G**) Input/output voltage curve without external stimulus. (**H**) Response consistency test of BPPS. The response results of six BPPSs under five half-sinusoidal wave impulses at 1 Hz are obtained.

The BPPSs were fabricated using microelectromechanical systems (MEMS) technology. The detailed fabrication process of the BPPS is described in Materials and Methods and illustrated in figs. S30 and S31. Figure 3A shows a typical plot of the relative change in voltage versus pressure of the BPPS, where *V*_0_ and Δ*V* represent the initial voltage and voltage change, respectively. The sensor shows a high sensitivity of 65.56 mV/V per kPa with a high determination fitting coefficient of 0.99934, laying the foundation for high-resolution applications of BPPS in aerodynamics, spatial positioning, and health care fields. A fitted straight line with a fixed intercept of 0 V is used as the reference curve for the calculation of the sensor’s nonlinearity to Eq. 1. As shown in Fig. 3B, within the range of 0 to 500 kPa, the nonlinearity of the sensor is less than 2.9%

$$e_{\text{NL}}=\pm \frac{∆Y}{Y_{\text{max}}}$$ (1)

*∆Y* is the difference between the actual output curve and the reference curve at the same pressure, and *Y*_max_ is the full-scale output (*35*, *36*).

To verify the accuracy and response of the BPPS, three types of pressure waveforms were applied onto the sensor membrane using a custom-designed pressure stimulation platform (fig. S32). These comprised sine, square, and triangular waves with an amplitude of 90 μm and a frequency of 1 Hz (Fig. 3C). The BPPS exhibited rapid responses to the detected pressure (square wave; Fig. 3D), which took ~10 ms before reaching the steady phase. In addition, the sensor recovered to the initial status within 4 ms after the pressure was removed. The negative terminals of the power supply and negative output were grounded together to achieve ultrahigh data acquisition rates and to mitigate noise from the power supply. Furthermore, the durability test was conducted to assess the long-term stability of sensors under continuous stimulations. It is found that the sensor exhibits high stability and reproducibility in response to cyclic loading, even after 20,000 cycles (Fig. 3E). Three sections of the curve, including the early stage (2501 to 2505), middle stage (10,001 to 10,005), and late stage (17,501 to 17,505) were intercepted and displayed separately, with almost no change in response amplitude and waveform. The peak points of the cyclic curve were extracted using the findpeaks function in MATLAB, and a survival prediction curve was obtained by performing linear fitting. This process is based on the degradation theory from reliability engineering to predict the life span of MEMS sensors (*37*, *38*). The linear fit result yielded a high correlation coefficient (*R*^2^ = 0.946), indicating a strong linear relationship between the number of cycles and the signal amplitude degradation from the linear regression model. It can be forecasted that the BPPS exhibits a response variation of 1%, which necessitates more than 0.6 million cycles for completion under the given stimulus. This indicates that BPPS has exceptional performance stability, with the potential for long-term and real-time monitoring applications.

On the basis of the fundamental tenets of Bernoulli’s principle, heightened flow velocities prompt a concomitant reduction in pressure. A discernible disparity in pressure manifests between its top and bottom surfaces as the airflow passes over the BPPS’s membrane. This discrepancy increases linearly with escalating flow velocities, thereby engendering prospects for its application within human respiratory monitoring. A self-constructed airflow stimulation testing system has been assembled, comprising the controllable airflow stimulation (excitation section) and real-time recording of BPPS output (testing section) (fig. S33). This system allows for manipulating the amount of airflow and the timing of the opening and closing of airflow. The BPPS response to the airflow velocity was a change in voltage, closely resembling the ramps and constant velocity holds of the airflow cycling over nominal velocities ranging from 0 to 6.96 m/s (fig. S34). Furthermore, we investigated the response of BPPS to the same amplitude but different frequencies of airflow (fig. S35). However, the limitations of the self-constructed testing platform precluded the conduct of experiments on airflow frequencies above 2.5 Hz. The durability performance of BPPS is also crucial for applications such as respiratory monitoring and near-body flow field perception for intelligent robots, as it provides real-time and long-term data (fig. S36). To deal with certain extreme situations, such as when sensors are subjected to continuous stimulation from high-velocity airflow, it is essential to conduct antidestructive (maintain the sensor’s sensing performance while minimizing damage to its basic sensing structure) performance testing. The detection limit of BPPS was validated using a strong airflow of 28.4 m/s. Following exposure to 206 strong pulse impacts, BPPS suffered from failure and damage (fig. S37). In addition, to investigate the sensor’s responsiveness to weak signals, a high-speed camera was used to capture the dynamic process of BPPS to a droplet of water (0.0053 g) produced from a 1-ml syringe. The electrical signal output was measured to change by 1 mV from 4.2 to 5.1 s, and after removing the droplet, the signal returned to its original level (fig. S38).

The commercialization of sensors necessitates the capacity for large-scale fabrication while ensuring stable performance parameters. Profit from the advanced maturity of MEMS technology, the sensors fabricated in this study demonstrated exceptional interchangeability when 16 randomly selected samples of the 180 were subjected to resistance uniformity, input/output voltage uniformity, and response uniformity tests. Figure 3F shows that numerical values of piezoresistors for 16 randomly selected samples are nearly identical, with fluctuations less than 0.003%, which is conducive to the balance of the Wheatstone bridge under no stimulation. Voltage-ampere characteristic testing was conducted on the sampled BPPS, yielding the output response at input voltages of 1, 3, 5, 7, 9, 11, 13, and 15 V (Fig. 3G). Under the half-sine pressure wave at 1 Hz, experiments were conducted on six sensor samples with highly consistent responses for potential large-scale production (Fig. 3H and fig. S39). When compared with pressure sensors in published works, our pressure sensor is the only one to attain all three key performances simultaneously: high sensitivity, high linearity, and wide measurement range (figs. S40 and S41 and tables S3 and S4).

### Flow regime detection and identification performance

**Fig. 4.**
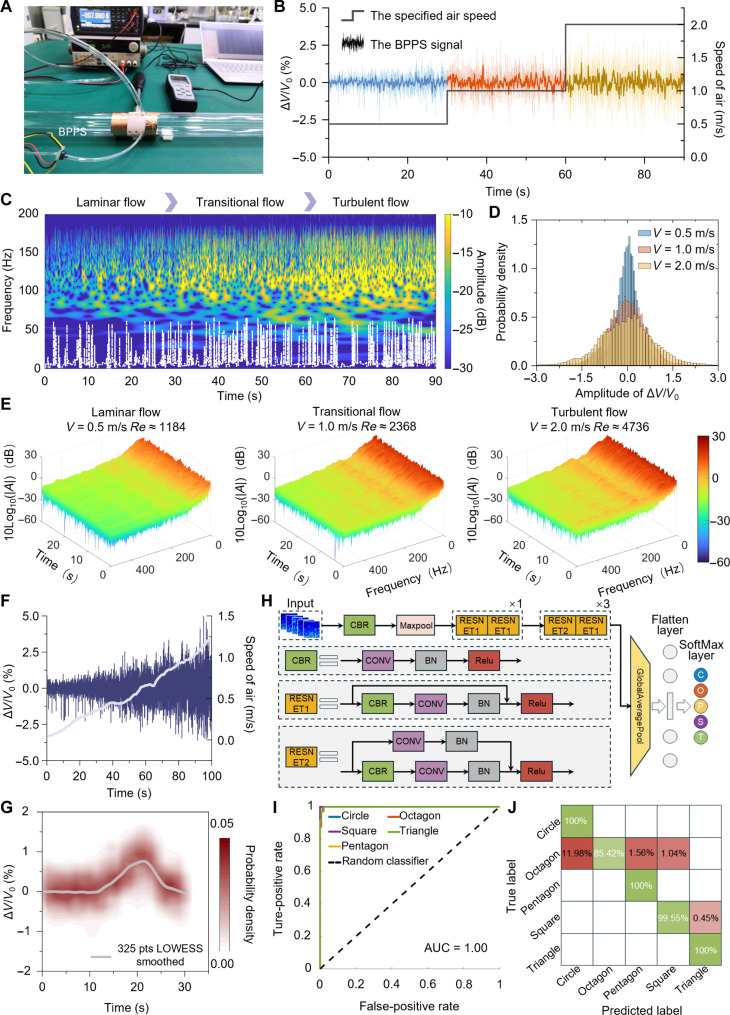
Flow regime detection and identification performance. (**A**) Experimental setup and acquisition circuit system. (**B**) The BPPS response curves under laminar (0.5 m/s), transitional (1.0 m/s), and turbulent (2.0 m/s) flow. (**C**) The instantaneous frequency (white dashed line) and amplitude (color mapping) of the three flow regime signals. (**D**) The amplitude probability density distribution. (**E**) Three flow regime signal’s time-frequency spectrograms (short-time Fourier transform). (**F**) The BPPS response curves under flow velocity ranging from 0.1 to 2.35 m/s. (**G**) Close-range testing. pts, points; LOWESS, locally weighted scatterplot smoothing. (**H**) The ResNet18 deep learning network structure. RESNET, residual network; CONV, convolution; BN, batch normalization; Relu, rectified linear unit. (**I**) Five-signal classification receiver operating characteristic curves. AUC, area under the curve. (**J**) The confusion matrix of the validation results.

The airflow field is one of the critical physical domains for real-time interaction with the environment. Precise detection and analysis of flow regime information enable robots to better interpret and respond to changes in their environment, strengthening their adaptability and thereby improving their operational efficiency and reliability in challenging scenarios. The experimental setup of the wind tunnel, which has a length of 37 mm with a rectifying microgrid at the upstream end to provide a stable and uniform flow field, is presented in Fig. 4A. A commercial anemobarometer was used to measure the free stream velocities and wind pressure. In particular, to minimize the impact of the pressure acquisition section on airflow, a 0.45-mm-diameter needle was mounted on the wind tunnel wall and connected to the external BPPS, and its bottom is linked to a constant pressure source (1 atm) to ensure isolation from environmental interference.

The BPPS can detect the flow regime and clearly distinguish laminar flow, transitional flow, and turbulent flow. Within the wind tunnel maintained at 25°C, the flow fields of these three different flow patterns are generated, corresponding to wind speeds of 0.5 m/s [Reynolds number (*Re*) ≈ 1184], 1 m/s (*Re* ≈ 2368), and 2 m/s (*Re* ≈ 4736), respectively. The pressure signals of the wind tunnel center flow field under each flow regime were collected by BPPS. The data obtained is illustrated in Fig. 4B, where the darker signal curves represent the smoothed data processed via the adjacent averaging method. Figure 4C shows the instantaneous frequency (white dashed line) and amplitude (color mapping) of the three flow regime signals calculated using wavelet transform. In the laminar flow regime, high-frequency signals (above 50 Hz) are infrequent and characterized by relatively low amplitudes (−20.92 dB). As the flow transitions into the critical flow regime, there is a marked increase in high-frequency signals, indicating increased fluctuations in the pressure signals, accompanied by a further increase in amplitude (−14.82 dB). In the turbulent flow regime, although the high-frequency components within the signals do not increase, the amplitudes of these signals continue to increase (about −9.08 dB), which is consistent with the observed characteristics of real flow phenomena.

Furthermore, the probability distribution of the signal amplitudes was analyzed, unveiling pronounced disparities among the three categories of signals, as graphically depicted in Fig. 4D. To comprehensively analyze the flow regime detection capability of BPPS, the short-time Fourier transform was used to calculate time-frequency spectrograms of pressure fluctuation signals across three distinct flow regimes (Fig. 4E). The color mapping reflects the power distribution of signals at different time and frequency domains. Compared to the time-frequency characteristic of laminar flow, the transitional flow signal’s amplitudes in the high-frequency range above 100 Hz rise obviously. For the turbulent flow signal, its time-frequency spectrogram reveals an increase in low-frequency signal amplitude besides the disorderliness of the high-frequency part. This observation aligns with the result of fluid dynamic analysis, confirming the reliable capability of our BPPS to accurately detect flow regime information.

To quantitatively evaluate BPPS’s flow detection capability of the BPPS, its output responses were measured while precisely controlling flow velocity in the wind tunnel, varying from 0.1 to 2.35 m/s (Fig. 4F). With the continuous increase of flow velocity in the wind tunnel, the output pressure signal of the BPPS changes accordingly. When the sensor is fixed on the displacement table to approach a wall at a speed of 2 m/s, the sensor signal demonstrates the detection potential of the BPPS for close distance (Fig. 4G). As the distance to the wall decreases, the output signal reaches up to 0.7%, which is comparable to the signal response observed in a 0.5 m/s flow field.

Compared with traditional biological optical recognition (which requires specific lighting conditions) and machine radar detection (which involves a complex system), the near-body flow field perception has many advantages including rapid perception, strong environmental adaptability, and low energy consumption. However, practical applications have been hampered by the limited detection accuracy of sensors and the challenges associated with analyzing pressure fluctuation data. Leveraging BPPS’s high-precision flow-field detection capability with the ResNet18 deep learning network, we developed a scorpion-inspired near-body flow field perceptual system (Fig. 4H) to detect and identify approaching object types based on the pressure fluctuations captured by BPPS in the near-body flow domain (*39*, *40*). The ResNet-18 model was trained by using our dataset of 1056 samples, with 70% for training and 30% for validation, while we also optimized the training process, including learning rate adjustments. For possible future on-board implementation, possible model optimization techniques could be used such as pruning, quantization, or be transitioned to lighter architectures like MobileNet or TinyML to reduce computational overhead. Five objects of different shapes (circle, octagon, pentagon, square, triangle, and all shapes are inscribed within a circle of 10-mm radius) approach the BPPS at 2 m/s to generate pressure fluctuations in the surrounding flow field. These fluctuation signals are converted into time-frequency images of size 224 × 224 × 3 using wavelet transform and fed into a ResNet-18 network for analysis. A dataset comprising 1056 time-frequency images, corresponding to the five objects, is constructed for network training and learning. Figure 4I shows the receiver operating characteristic curve of the system, where the area under the curve for all five object shapes is equal to 1. This indicates that the system demonstrates good classification performance for five object categories.

The confusion matrix of the validation results is shown in Fig. 4J. The recognition accuracy for the circle, pentagon, square, and triangle exceeds 99.5%. However, for the octagon, particularly when distinguishing between the octagon and the circle, an error rate of 11.98% was observed. This may be attributed to the shape similarity between the two objects, which leads to comparable flow field pressure fluctuations. Hence, by integrating the wavelet transform algorithm and the ResNet18 deep learning network, the system enables the identification of various flow field pressure fluctuations, offering promising potential for applications in the near-field flow perception and recognition domain for intelligent robots.

### Demonstration of near-body flow field perception for intelligent robots

Scorpions, as nocturnal animals, rely solely on mechanoreceptors (slit and trichobothria sensilla) for survival behaviors including evading predators and hunting (*41*–*44*). Unidirectional trichobothria and slit sensilla are the two key components of the fused out-of-plane and in-plane sensory input used by scorpions to monitor the surrounding environment field. We took inspiration from such neurophysiological evidence and postulated a sensory mechanism for scorpion *H. petersii* that can explain behavioral experiments that scorpions invisibly avoid predators and perform hunting (fig. S42). We demonstrate how nearby objects may be detected by scorpions using the near-body flow field fluctuations during moving object intrusion, which is modulated in response to the flow field detected by their mechanosensory system.

**Fig. 5.**
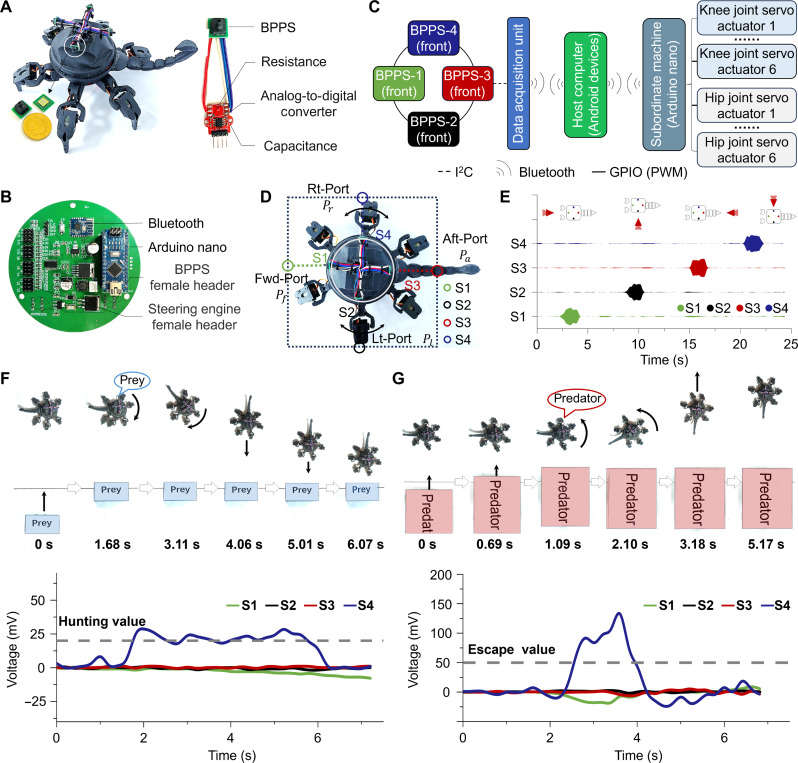
Near-body flow field perception for intelligent robots. (**A**) The scorpion-inspired hexapod walking platform equipped with four BPPSs. (**B**) The PCB design of the bioinspired near-body flow field perception robot. (**C**) Schematic diagram of the bioinspired robot’s functional components for near-body flow field perception. The system mainly consists of four BPPSs, a microcontroller unit (Atmega328P), a Bluetooth module (hc-08), and 12 servo actuators (mg90s). GPIO, general purpose input/output; PWM, pulse width modulation. (**D**) The arrangement of the four BPPSs is configured to maximize the detected pressure deltas when an object approaches the bioinspired robot. Fwd, forward; Lt, left; Rt, right. (**E**) The kite diagram illustrates the response of the BPPSs when subjected to airflow at 0.5 m/s from various directions, as indicated by the red arrows. (**F**) Signal response of hexapod walking platform toward prey simulator (small cardboard box) approaching at 0.25 m/s. (**G**) Signal response of hexapod walking platform toward predator simulator approaching at 0.25 m/s.

To demonstrate how mechanosensory flow field monitoring can be used in collision avoidance systems, we have developed a small hexapod walking platform with a bioinspired sensor package containing four BPPSs to distinguish large and small objects by the modulation of a flow field (Fig. 5A). It is lightweight, power efficient with high-precision, and no additional emission of light or electromagnetic radiation. Custom circuits and algorithms are designed to identify near-body flow field fluctuations based on BPPS readings (Fig. 5B and fig. S43). Figure 5C presents the modular design of the hexapod walking platform electronic system for the collection of complex flow field pressure, wireless transmission, and self-control drive. The raw data of near-body flow field fluctuations were obtained by the data acquisition unit and converted into digital signals via 24-bit analog-to-digital converters. These converted signals were sent through the wireless transceivers (HC-08) to the host computer for processing. After the decision-making process, a subordinate machine controlled the motions of the six knee joint servo actuators and six hip joint servo actuators. A customized program based on the software Android Studio was used for data logging and advanced human interface applications. The device, like scorpions, is most sensitive if sensors are mounted at locations experiencing the greatest changes in the flow field when approached by different-sized objects. Nearby objects distort the flow field around the body, making surface detection simple, direct, and robust (Fig. 5D).

Because of dynamic pressure being proportional to the square of flow velocity, the same physical phenomenon underpins the sensing capability. Figure 5E demonstrates the fluctuation of output data from four sensors on the scorpion-like hexapod walking platform when facing a square box (48 cm by 33 cm by 33 cm) approaching from four directions sequentially (0.5 m/s). Regardless of which direction the object approaches, the corresponding BPPS can sensitively monitor pressure fluctuations in the near-body field, with a consistent response of 145 ± 18 mV across all four directions, providing data reference for the device behavior. Inspired by the scorpion’s synaptic thresholding mechanism for computing escape decisions, we have designed a simple algorithm as a proof-of-concept demonstration and defined hunting value (*a* = 20) and escape value (*b* = 50) (fig. S44). If the numerical values of all four BPPS are less than the hunting value, then the hexapod walking platform remains upright. As soon as the value *p_i_* of one BPPS falls between the hunting and escape values, the hexapod walking platform adjusts its head direction to move toward that direction, mimicking the behavior of hunting the prey. If the sensor value exceeds the escape value, the device moves away from the direction of that sensor to mimic the behavior of avoiding predators.

We used differently sized cardboard boxes as analogs for predators and prey, pushing them toward the hexapod walking platform at different speeds using a lever. Figure 5F shows that when the small cardboard box (20 cm by 12 cm by 15 cm) as a prey simulator approaches the right side of the hexapod walking platform at a speed of ~0.25 m/s, the S4 sensor detects pressure fluctuations in the near-body flow field and outputs a measurement value of 22.6 ± 4.2 mV. For the time being, Atmega328P accesses the data acquisition unit twice continuously in a traversal manner, and the value of S4 is greater than the hunting value but less than the escape value. At 4.06 s, the hexapod walking platform adjusts its head toward the direction of the simulated prey and makes a close approach within the next 2 s. Figure 5G shows the hexapod walking platform’s sensor response toward the predator simulator (large box). When the natural enemy object approached the right side of the hexapod platform at a speed of 0.25 m/s, S4 instantly exhibited a strong output of 100.8 ± 32.3 mV, immediately moving away from the enemy object.

Unlike conventional intelligent robots, which have near-body perception with vision-based techniques such as infrared, visible light, and laser, the scheme demonstrated in this work based on near-field flow fluctuations can distinguish objects of different speeds and sizes in the near-body field by four BPPSs. By mimicking the neurological sensory mechanism of scorpions, the behaviors of predator avoidance and hunting prey of the prototype hexapod walking platform have been demonstrated. This strategy is direct, concise, low cost, and robust for controlling the movement of mobile devices by sensing airflows to detect dynamic changes in the surrounding such as object motions, obstacle positions, or environmental disturbances. Algorithms have been developed to analyze flow field characteristics, such as velocity, direction, and turbulence for near-field (centimeter-to-meter scale) flow variations, while conventional vision-based detections often detect objects for mid-to-long range (meter–to–hundred-meter scale) with high computation power consumption. Furthermore, flow-based detection relies on the physical properties of fluid media and is not affected by lighting conditions, such as fog, smoke, darkness, or occlusions, which can serve as a new tool for near-field robotic perception applications. For future perspective, the integration of BPPS on humanoid robot surfaces for near-body perception is a promising direction after addressing some engineering challenges, such as conformal adaptation to curved surfaces, signal cross-talk, and signal decoupling for standardized and modular sensing units (*45*, *46*).

## DISCUSSION

Conventional pressure sensors typically struggle to achieve high sensitivity and high linearity over a broad sensing range. Here, inspired by the stress concentration effect and the nonlinear energy conversion suppression mechanism of the scorpion mechanosensory, our team has proposed a strategy to enhance the sensitivity and linearity synergistically of silicon micromachined pressure sensors by constructing stress traps and flexural suppression structures for a balanced synergy between sensitivity and linearity. The prototype BPPS simultaneously exhibits a high sensitivity at 65.56 mV/V per kPa, good linearity of 0.99934 over an ultrawide linear pressure range of 0 to 500 kPa, along with fast response at 72 ms, fast recovery at 16 ms, and exceptional durability for more than 20,000 cycles. Together with the coupled mechanical-electric simulations, the versatile modulation of structural parameters in the sensor design provides a viable design toolkit to break the limitation in the trade-off between the sensitivity and linear sensing range. The potential of the high-performance BPPS is highlighted and demonstrated through proof-of-concept trials. These include showcasing its capability in approaching object type recognition with more than 85.42% accuracy and enabling active collision avoidance of a custom-built hexapod robot for intelligent locomotion. Our bioinspired structural design strategy to achieve high sensitivity and linearity over a wide range is a key advancement shift in the field of sensor design. This approach could also be applied to other types of pressure sensors, such as piezocapacitive sensors.

## MATERIALS AND METHODS

### Materials

The scorpions (*H. petersii*) were collected from Anhui Province, China. Scorpions were put in the feeding box with wet coconut soil, and the temperature in the box was kept between 25° and 30°C. In addition, the appropriate bread insect was given once a week. All animal experiments were performed in accordance with the procedures and protocols approved by the Ethics Committee for the Use of Experimental Animals of Jilin University (Changchun, China).

### Morphology characterization

The external morphology of the scorpion slit and trichobothria sensilla was studied using a digital microscope with a super depth of field (VHX-5000, Japan) and Tungsten filament scanning electron microscope (TESCAN VEGA GMS, Czech).

### Semithin section

First, scorpion walking legs were immersion-fixed in glutaraldehyde (4%) in phosphate buffer (0.1 M) overnight. Next, walking legs were immersed in osmium tetroxide (1%) in cacodylate buffer (0.1 M) for 2 hours. Then, the tissues were dehydrated in a progressive ethanol series from 50 to 100% before being embedded in an epoxy resin. Last, semithin sections were cut using a microtome (LEICA EMUC7, Leica Company, Germany), stained with 1% toluidine blue, and subsequently examined using a microscope (X71, Olympus, Japan).

### Compression tests and DIC

Using commercial 3D printing technology, we prepared three scaled slit sensillum resin samples with dimensions of 10 cm and a seam width of ~2 mm. After printing, the samples underwent secondary curing for 30 min using a 405-nm ultraviolet light source. All samples were then tested in a quasistatic compression mode by applying a load that mimicked the actual operating conditions of slit sensillum at a rate of 0.5 mm/min with a universal testing machine (Instron; model 5948). The videos of the compression tests were recorded with an Amscope camera with a frame rate of 500 f/min, which was used for DIC analysis. The strain fields were calculated using VIC-3D (Correlated Solutions) with an incremental algorithm and a subset size of 35 pixels.

### 3D μ-CT scanning for trichobothria sensilla

Through micromanipulation, a biologically active trichobothria sensilla tissue (~2 mm) was extracted and subsequently fixed onto a testing rod using a light-curing adhesive. Submicrometer 3D imaging of the sample was conducted using an x-ray microtomography system (Zeiss X-ray Microscopy, Xradia 620 Versa). A monochromatic x-ray beam with an energy of 80 keV and a power of 30 W was used for the imaging process. The acquired .txm files were directly processed using onboard CT data processing software provided by Zeiss.

### Mechanics simulations

To assist in the design and optimization of BPPS, we developed different structural models in COMSOL to quantitatively predict the volumetric strain behavior and stress concentration of SOI silicon wafer at an imposed constant pressure *P* = 1 kPa. In the design of the model’s shape, the size of the dimensions in the analysis process is the same as the actual size of the dimensions. Specific material parameters can be viewed in table S1. The grid is delineated by an adaptive mesh refinement process in the solid mechanics module, which is intended to ensure the accuracy of the results. Fixed constraints on silicon-glass anodic bonding area were exploited by applying pressures similar to the actual working conditions to the surface of the network specimen.

### Fabrication of BPPSs

The silicon manufacturing process was processed at the Suzhou Institute of Nano-tech and Nano-bionics. BPPS is fabricated using silicon micromachining technology on a 20-μm-thick SOI silicon wafer. The preparation process mainly includes the manufacture of piezoresistors, etching and structural release of biomimetic structures, metal film sputtering and lead formation, and silicon glass anodic bonding. Using silica and photoresist as mask layers for boron ion implantation, the injection energy is 40 keV, with a heavy doping dose of 2 × 10^16^ cm^−3^ and a light doping dose of 1.6 × 10^16^ cm^−3^. A layer of Au thin film with a thickness of 0.2 μm is sputtered on the silicon wafer using magnetron sputtering equipment. The bonding temperature is 305°C, with a center voltage of 1000 V and an edge voltage of 800 V.

### Sensor characterizations

To characterize the electromechanical performance of BPPS, a self-constructed airflow stimulation testing system has been assembled, comprising the controllable airflow stimulation (excitation section) and real-time recording of BPPS output (testing section) (fig. S31). A constant current voltage source (K3205D, MCH, China) is used to provide a stable voltage of 5 V for BPPS. The electrical signals of the sensor were read by a digit multimeter (Keysight, 34465A, USA) with Keysight BenchVue software. To sensitively capture changes in sensor signals, a resolution of 0.01 s was adopted.

## Supplementary Materials

### The PDF file includes:

Figs. S1 to S44 Notes S1 to S4 Tables S1 to S4 Legend for movie S1
