# Supplementary material for: Hypersensitive pressure sensors inspired by scorpion mechanosensory mechanisms for near-body flow detection in intelligent robots
Source: Sci Adv. 2025 Aug 20;11(34):eady5008. doi: 10.1126/sciadv.ady5008 (PMC12366690; doi:10.1126/sciadv.ady5008)
Supplement: Supplementary file 1 — Figs. S1 to S44 Notes S1 to S4 Tables S1 to S4 Legend for movie S1 [file sciadv.ady5008_sm.pdf]

Science Advances
AAAS

Supplementary Materials for

Hypersensitive pressure sensors inspired by scorpion mechanosensory mechanisms for near-body flow detection in intelligent robots

Pinkun Wang *et al.*

Corresponding author: Bo Li, boli@jlu.edu.cn; Shichao Niu, niushichao@jlu.edu.cn;
Zhiwu Han, zwhan@jlu.edu.cn; Liwei Lin, lwlin@berkeley.edu

Sci. Adv. **11**, eady5008 (2025)
DOI: 10.1126/sciadv.ady5008

The PDF file includes:

Figs. S1 to S44
Notes S1 to S4
Tables S1 to S4
Legend for movie S1

Other Supplementary Material for this manuscript includes the following:

Movie S1

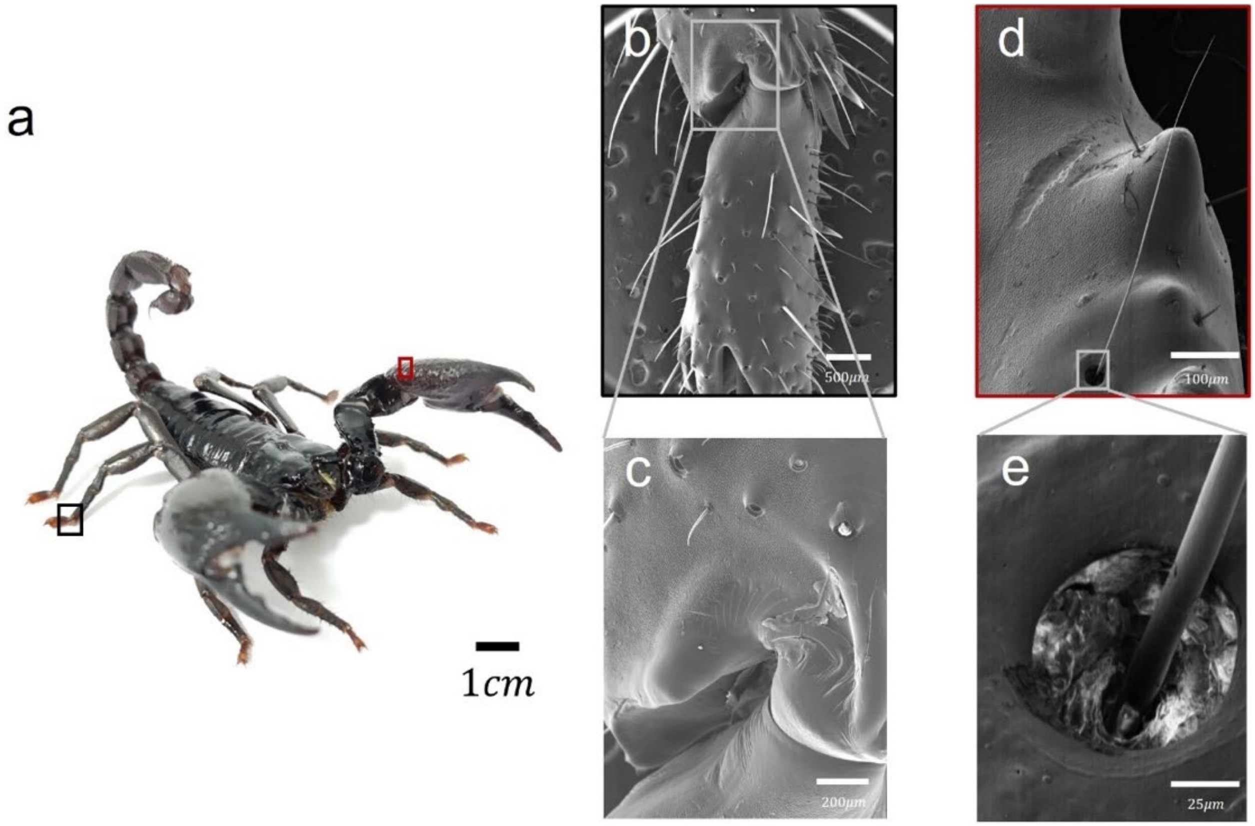

Fig. S1 | Scorpion mechanosensory sensilla. a, Optical image of scorpion (*Heterometrus petersii*). **b**, The SEM image of slit sensilla. They are located on the tarsus of the scorpion's foot, specifically at the distal end of the basitarsus. **c**, The enlarged SEM image of slit sensilla. **d**, The SEM of trichobothria sensilla. They are located on the chela of the scorpion, with approximately 45. **e**, The end of trichobothria sensilla.

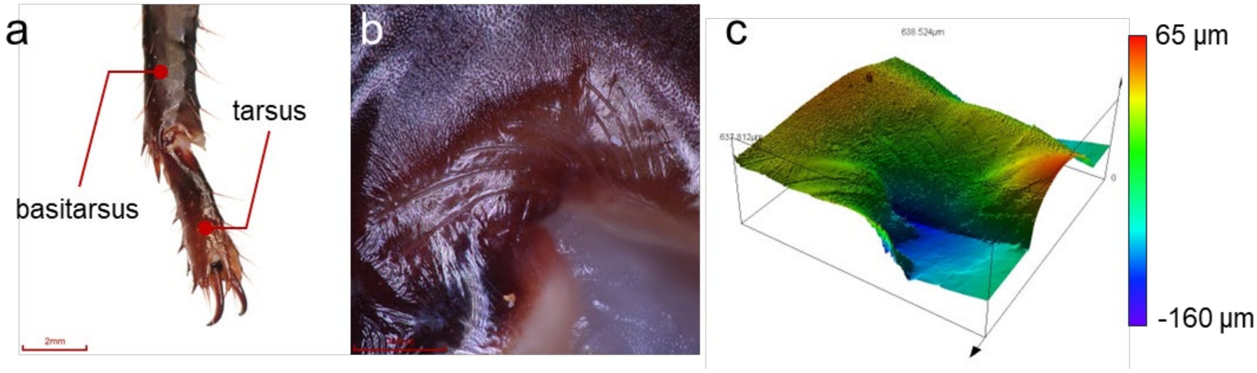

Fig. S2 | The images of slit sensilla. (a) Optical image of the scorpion's walking leg. (b) Optical image of slit sensilla. (c) The three-dimensional image of slit sensilla.

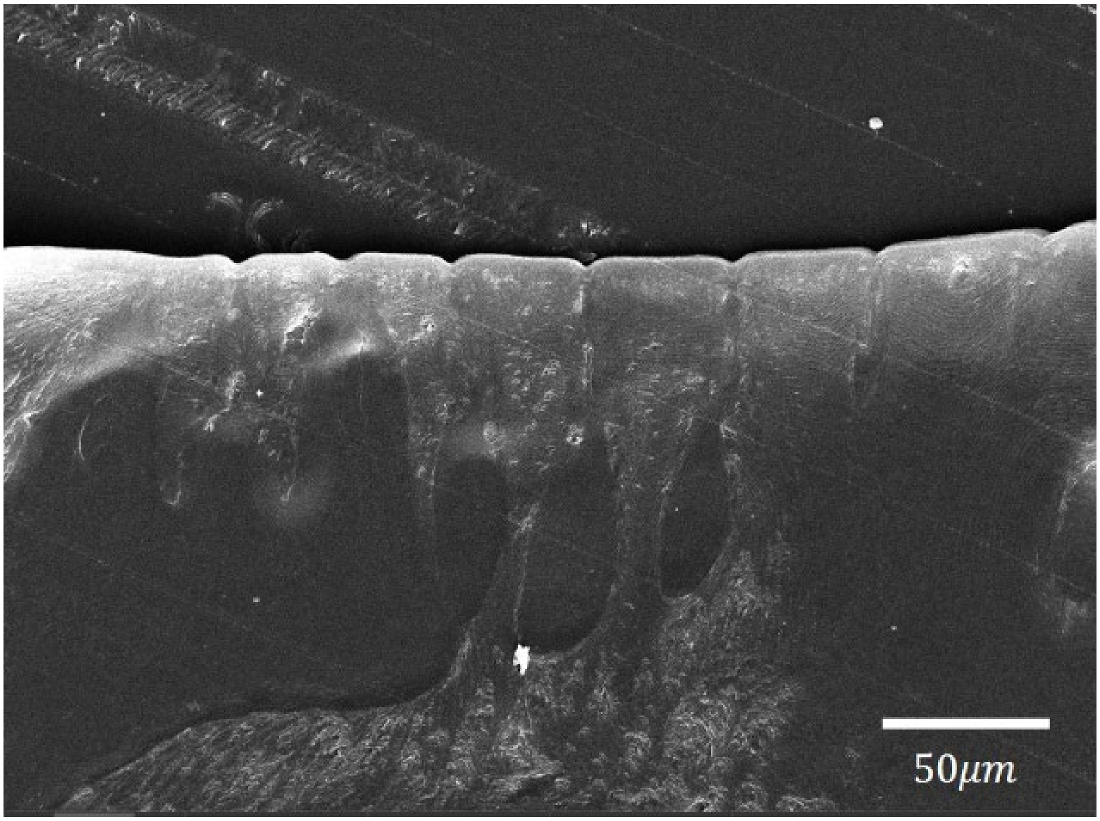

Fig. S3 | Semithin section of scorpion slit sensilla made in the fresh state. The cross-sectional morphology of the slit sensilla was observed under dehydration conditions, revealing that the slit sensilla is composed of thick blocks constituting the main body and thin cuticular membranes covering each crack-shaped slit.

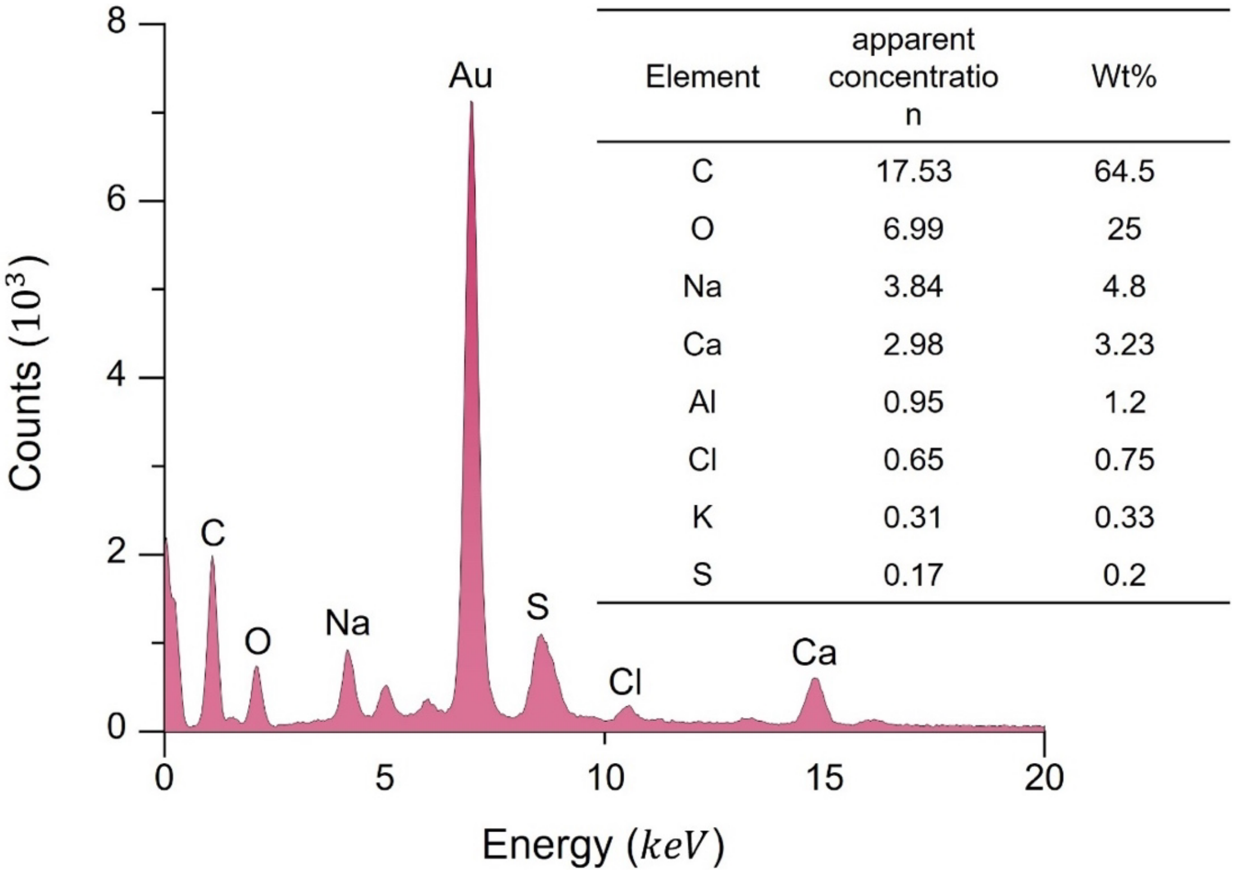

Element	apparent concentratio n	Wt%
C	17.53	64.5
O	6.99	25
Na	3.84	4.8
Ca	2.98	3.23
Al	0.95	1.2
Cl	0.65	0.75
K	0.31	0.33
S	0.17	0.2

Fig. S4 | EMSA/MAS spectral data of scorpion slit sensilla's tissue. The biological tissue of scorpion slit sensilla is mainly composed of elements such as C and O, accounting for approximately 89.5%.

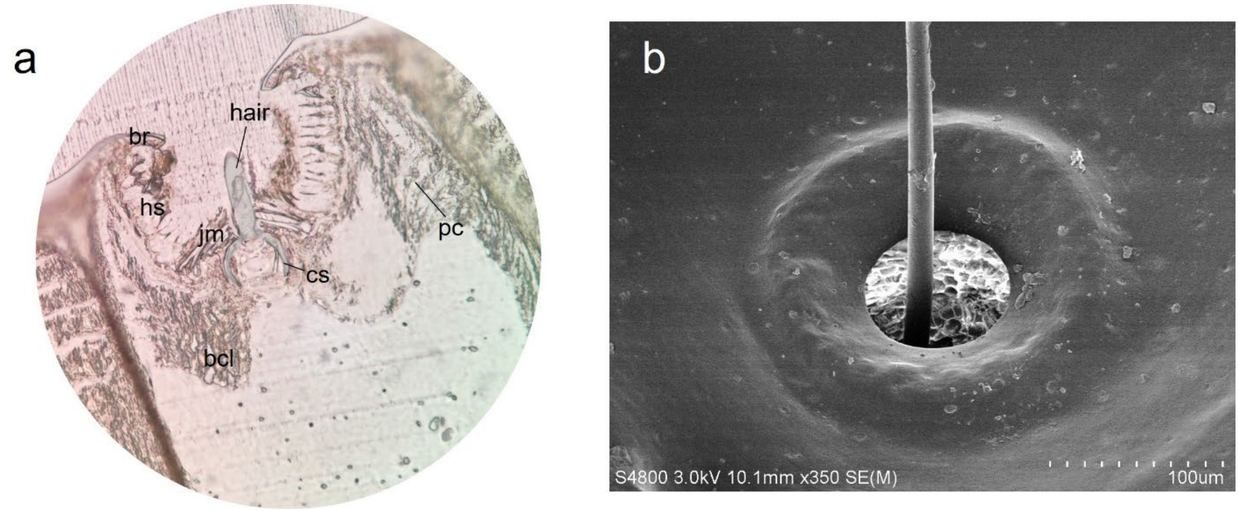

Fig. S5 | Trichobothrium of the scorpion *Heterometrus petersii*. a, Longitudinal section of a trichobothrium perpendicular to the hair's plane of oscillation. Boundary load conditions. The claw-like structure(cs) appears symmetrical. Many pore canals (pc) traverse the endocuticle. br, rim of the bothrium; hs, honeycomb structure; jm, joint membrane; bcl, basal cell layer. **b**, Scanning electron micrographs of trichobothria. Long and slender hair shafts protrude from a "bothrium", a cup-like cuticular cavity encircled by an elevated rim.

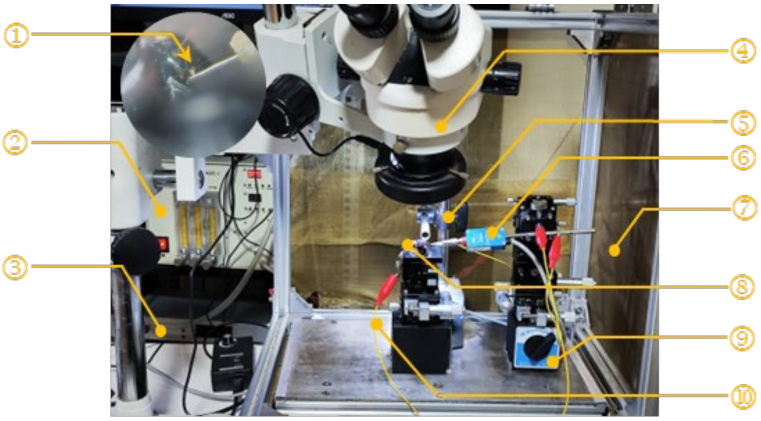

1. trichobothria sensilla
2. Stimulating airflow generator
3. Four channel signal collector
4. Microscope
5. Airflow outlet (8mm)
6. Probe (+)
7. Faraday cage
8. Pedipalp
9. 3-DOF displacement table
10. ground wire (-)

Fig. S6 | Gas stimulation electrophysiological testing system.

Note S1. Geometric shapes of membrane

In pressure sensors, a square membrane is chosen because of its higher stress when compared with rectangular and circular ones in the same conditions. On the basis of the theory of elasticity, the maximum stresses of the square, rectangular, and circular membranes, respectively, are given as follows:

$$\sigma_{sm} = 0.308\left(\frac{L}{H}\right)^2 (1-\mu^2)P \tag{1}$$

$$\sigma_{rm} = 0.383\left(\frac{B}{H}\right)^2 (1-\mu^2)P \tag{2}$$

$$\sigma_{cm} = 0.75\left(\frac{R}{H}\right)^2 (1-\mu^2)P \tag{3}$$

where σ_{sm}, σ_{rm}, and σ_{cm} are the maximum stresses for the square, rectangular, and circular membranes, respectively,

P——the applied pressure

H——the thickness of the membrane

μ——the Poisson ratio

L——the side length of the square membrane

B——the width of the rectangular membrane

R——the radius of the circular membrane

By assuming that they all have the same membrane thickness H and identical applied pressure P, and additionally that, L is 1.2 times B and 2 times R, the following relationship can be obtained:

$$\sigma_{sm} = 1.16\sigma_{rm} = 1.64\sigma_{cm} \tag{4}$$

which means ~15% and ~60% higher stress can be achieved by utilizing square membrane rather than the other two types.

Using the COMSOL solid mechanics module for simulation and further verification, the stress cloud diagrams in the following three figures can intuitively show that square membranes have higher *von Mises* stress than rectangular and circular membranes at an imposed constant pressure p=1 kPa.

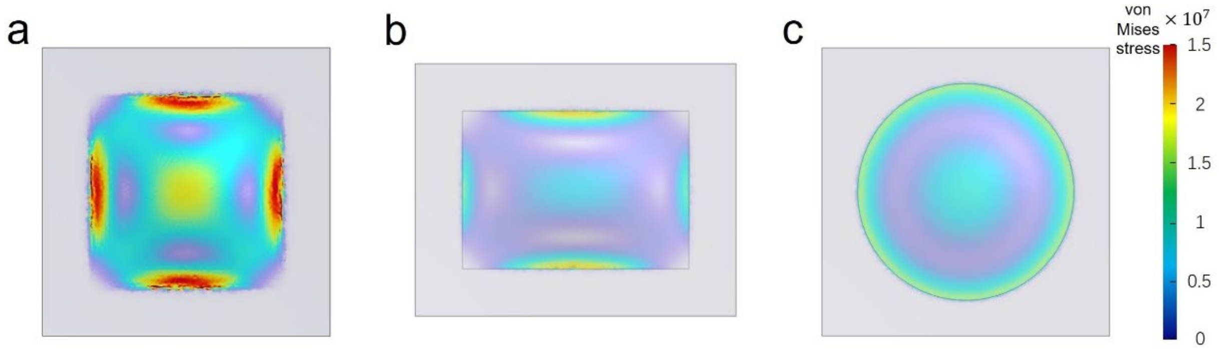

Fig. S7 | Stress cloud map extracted from linear FE analysis. **a**, square membrane; **b**, rectangular membrane; **c**, circular membrane.

Note S2. Wheatstone bridge circuit

The Wheatstone bridge is a detection method used in MEMS resistive pressure sensors to detect changes in piezoresistors. Usually, the most sensitive Wheatstone full bridge circuit is used, which improves full-range output and reduces zero temperature drift.

Four sets of piezoresistors are connected end-to-end, with two non-adjacent terminals used for voltage input and the other two for voltage output, forming an equivalent Wheatstone bridge. Due to the same manufacturing process of the four piezoresistors R, their resistance values are the same. When the silicon membrane is not subjected to other external pressures, the resistance values of the four piezoresistors do not change relatively, $R_{i1} = R_{i2} = R_{d1} = R_{d2} = R$. the output voltage of the bridge is 0; When a pressure difference is formed between the upper and lower surfaces of the membrane, it will cause deformation of the membrane, and the tension around the center can cause compression in the X direction and stretching in the Y direction in the area where varistors R_{i1} and R_{i2} are located, resulting in an increase in the resistance values of varistors R_{i1} and R_{i2}. Conversely, the tension around the center can cause a decrease in the resistance values of varistors R_{i1} and R_{i2}. Assuming that the resistance values of all four varistors change by ΔR, the relationship between the output voltage and the input voltage is:

$$U_{out} = \left(\frac{\Delta R}{R}\right) U_{in} = \left(\frac{\Delta \rho}{\rho}\right) U_{in} \tag{5}$$

Where ρ and $\Delta\rho$ represent resistivity and resistivity change respectively.

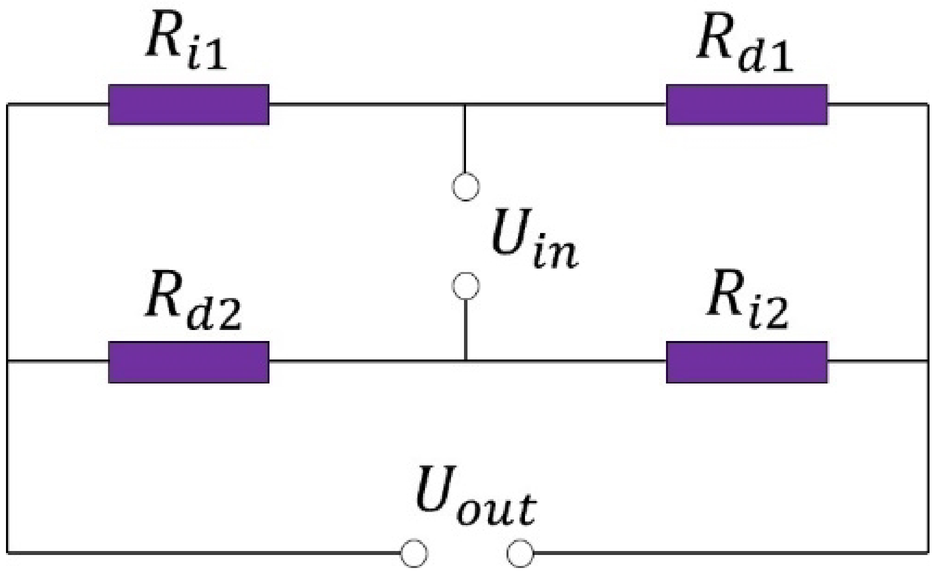

Fig. S8 | Wheatstone bridge circuit.

Note S3. Piezoresistive effect of piezoresistors on SOI silicon wafer

The piezoresistive effect of monocrystalline silicon refers to the change in resistance caused by changes in stress, which is essentially a change in electrical resistivity. Monocrystalline silicon belongs to the face-centered cubic crystal system. It has O_h point group symmetric structure and the resistivity tensor $[\rho]$ can be expressed as a column vector:

$$[\rho] = [\rho_1 \quad \rho_2 \quad \rho_3 \quad \rho_4 \quad \rho_5 \quad \rho_6]^T \tag{6}$$

Similarly, the stress tensor $[\sigma]$ inside monocrystalline silicon can also be expressed as:

$$[\sigma] = [\sigma_1 \quad \sigma_2 \quad \sigma_3 \quad \sigma_4 \quad \sigma_5 \quad \sigma_6]^T \tag{7}$$

The piezoresistive coefficient matrix $[\pi]$ of monocrystalline silicon can be expressed as:

$$[\pi] = \begin{bmatrix} \pi_{11} & \pi_{12} & \pi_{12} & 0 & 0 & 0 \\ \pi_{12} & \pi_{11} & \pi_{12} & 0 & 0 & 0 \\ \pi_{12} & \pi_{12} & \pi_{11} & 0 & 0 & 0 \\ 0 & 0 & 0 & \pi_{44} & 0 & 0 \\ 0 & 0 & 0 & 0 & \pi_{44} & 0 \\ 0 & 0 & 0 & 0 & 0 & \pi_{44} \end{bmatrix} \tag{8}$$

The relationship between the change in resistivity $\Delta\rho$ and the stress tensor σ and the piezoresistive coefficient π can be expressed as:

$$\Delta\rho = \pi\sigma \tag{9}$$

Specifically,

$$\begin{bmatrix} \Delta\rho_1 \\ \Delta\rho_2 \\ \Delta\rho_3 \\ \Delta\rho_4 \\ \Delta\rho_5 \\ \Delta\rho_6 \end{bmatrix} = \begin{bmatrix} \pi_{11} & \pi_{12} & \pi_{12} & 0 & 0 & 0 \\ \pi_{12} & \pi_{11} & \pi_{12} & 0 & 0 & 0 \\ \pi_{12} & \pi_{12} & \pi_{11} & 0 & 0 & 0 \\ 0 & 0 & 0 & \pi_{44} & 0 & 0 \\ 0 & 0 & 0 & 0 & \pi_{44} & 0 \\ 0 & 0 & 0 & 0 & 0 & \pi_{44} \end{bmatrix} \begin{bmatrix} \sigma_1 \\ \sigma_2 \\ \sigma_3 \\ \sigma_4 \\ \sigma_5 \\ \sigma_6 \end{bmatrix} \tag{10}$$

All piezoresistors are placed along the [110] direction on the N-type silicon (100) crystal plane of the SOI silicon wafer, as shown in Figure S7. The relationship between the relative resistance change caused by the piezoresistive effect of P-type varistors under stress is as follows:

$$\frac{\Delta\rho}{\rho} = \pi_l\sigma_l + \pi_t\sigma_t = \frac{\pi_{44}}{2}(\sigma_l - \sigma_t) \tag{11}$$

Where σ_l is the longitudinal stress of the piezoresistors, σ_t is the transverse stress of the piezoresistors, π_{44} is the shear piezoresistive coefficient, π_l is the longitudinal piezoresistive coefficient, and π_t is the transverse piezoresistive coefficient.

According to membrane theory, flexural deflection $\omega(x, y)$ of SOI silicon wafer can be expressed as:

$$\omega(x,y)=\frac{p}{24D}(a^2x^2-x^4)(b^2y^2-y^4) \tag{12}$$

Where p is the pressure applied to the silicon wafer, D is the bending stiffness of the membrane, a and b are half of the side length of the membrane, x and y are the horizontal and vertical coordinates from the four fixed boundaries, respectively.

The components σ_l and σ_t of stress can be expressed as:

$$\sigma_l=\sigma_x(x,y)=E\frac{\partial\omega}{\partial x}=E\omega(x,y)\frac{2a^2-4x^2}{a^2x-x^3} \tag{13}$$

$$\sigma_t=\sigma_y(x,y)=E\frac{\partial\omega}{\partial y}=E\omega(x,y)\frac{2b^2-4y^2}{b^2y-y^3} \tag{14}$$

Where E is the elastic modulus of the SOI silicon wafer.

Based on Eqs. (5), (11), (12), (13) and (14), the sensitivity (S) of the sensor then is given by

$$\begin{aligned} S\equiv\frac{\Delta U_{out}}{\Delta p}&=\frac{1}{2}\pi_{44}U_{in}\frac{\Delta(\sigma_l-\sigma_t)}{\Delta p} \\ &=A(x,y)B(x,y)\frac{1-v^2}{t^3} \end{aligned} \tag{16}$$

Wherein,

$$A(x,y)=\frac{2a^2-4x^2}{a^2x-x^3}-\frac{2b^2-4y^2}{b^2y-y^3}$$

$$B(x,y)=\frac{(a^2x^2-x^4)(b^2y^2-y^4)}{4}\pi_{44}U_{in}$$

Where v is the Poisson's ratio, t represents the thickness of the SOI silicon wafer.

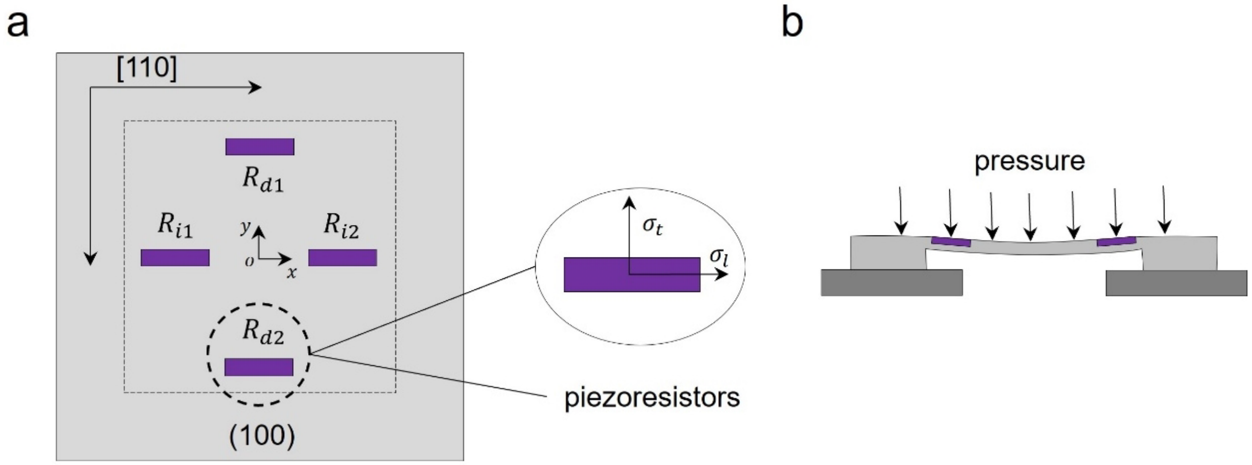

Fig. S9 | BPPS silicon wafer layout and sensing principles. **a**, Distribution diagram of piezoresistors on silicon membrane; **b**, membrane-typed piezoresistive pressure sensor detection principle diagram.

Note S4. The deterministic relationship between thin-film mechanics and performance

To demonstrate that a stress trap unit inspired by the slit sensilla of scorpions can enhance the sensitivity of a MEMS thin-film pressure sensor, and that a flexure suppression unit inspired by the trichobothria sensilla can improve sensor's linearity, we derive these effects using thin-film mechanics and the Föppl-von Kármán (FvK) equations. The theoretical analysis is conducted from an energy and stress-strain perspective, elucidating the mechanical mechanisms of both designs and their respective impacts on sensitivity and linearity.

a) Theoretical mechanism of the stress trap unit for enhancing sensor sensitivity

Consider a square MEMS membrane (side length $2a$, thickness h, fixed edges) subjected to uniform external pressure p, with deflection $\omega(x,y)$. A Wheatstone bridge is arranged on the thin membrane.

The deflection $\omega(x,y)$ satisfies the FvK equation:

$$D\nabla^4\omega - h\left(\sigma_x\frac{\partial^2\omega}{\partial x^2} + \sigma_y\frac{\partial^2\omega}{\partial y^2} + 2\tau_{xy}\frac{\partial^2\omega}{\partial x\partial y}\right) = p$$

Where $D = \frac{Eh^3}{12(1-v^2)}$ is the bending rigidity, and ∂x, ∂y, τ_{xy} are in-plane stresses.

Small deflection (linear regime):

For small deflections ($\omega \ll h$), stretching stresses (balloon effect) are negligible, and the governing equation simplifies to:

$$D\nabla^4\omega = p$$

Assume a deflection profile:

$$\omega \approx \omega_0(1-\frac{x^2}{a^2})(1-\frac{y^2}{a^2})$$

The maximum deflection at the center ($\omega_0 = \omega(0,0)$) is found by minimizing the potential energy $\Pi = U_b - W$, where:

Bending strain energy:

$$U_b = \frac{D}{2}\iint(\nabla^2\omega)^2dxdy$$

External work:

$$W = \iint p\omega dxdy$$

Using the variational method:

$$\omega_0 \propto \frac{pa^4}{Eh^3}$$

The in-plane strain is:

$$\epsilon_x = \frac{1}{2}(\frac{\partial\omega}{\partial x})^2,\ \epsilon_y = \frac{1}{2}(\frac{\partial\omega}{\partial y})^2$$

$$\frac{\partial\omega}{\partial x} \approx -\frac{2\omega_0 x}{a^2}(1-\frac{y^2}{a^2}),\ \epsilon_x \approx \frac{2{\omega_0}^2x^2}{a^4}(1-\frac{y^2}{a^2})^2$$

Piezoresistors are positioned near the edge region of the membrane, thereby:

$$x \approx a,\ y \approx 0$$

$$\epsilon_x \approx \frac{2{\omega_0}^2}{a^2} \propto \frac{p^2a^6}{E^2h^6}$$

Sensitivity:

$$S \approx \frac{\partial(\frac{\Delta V}{V})}{\partial p} \propto \frac{\partial\epsilon_x}{\partial p} \propto \frac{pa^6}{E^2h^6}$$

After constructing stress trap unit, the local stress $\sigma_x = E\epsilon_x/(1-\nu)$ is amplified several

times. Assuming the stress concentration factor is K, the local strain is:

$$\epsilon_{x,\ local} = K\epsilon_x \propto K\frac{pa^6}{E^2h^6}$$

Therefore, by constructing stress trap unit, the sensitivity of the sensor can be directly enhanced during the small deflection stage.

Large deflection (nonlinear regime):

For large deflections ($\omega \sim h$), stretching energy dominates, and $\omega \propto p^{1/3}$. The strain is:

$$\epsilon_x \propto \omega^2 \propto p^{2/3}$$

Sensitivity:

$$S \approx \frac{\partial \epsilon_{x,\ local}}{\partial p} \propto Kp^{-1/3}$$

Therefore, by constructing stress trap unit, the sensitivity of the sensor can be directly enhanced during the large deflection stage.

b) Theoretical mechanism of the flexure suppression unit for enhancing sensor linearity

For a thin film without flexure suppression unit under large deformation, the FvK equations indicate:

$$D\nabla^4\omega - h\left(\sigma_x\frac{\partial^2\omega}{\partial x^2} + \sigma_y\frac{\partial^2\omega}{\partial y^2}\right) = p$$

Stretching strain energy:

$$U_s = \frac{h}{2}\iint(\sigma_x\epsilon_x + \sigma_y\epsilon_y)dxdy,\ \epsilon_x = \frac{1}{2}(\frac{\partial\omega}{\partial x})^2$$

The strain is:

$$\epsilon_x \approx \frac{2\omega_0^2x^2}{a^4}(1-\frac{y^2}{a^2})^2$$

Average strain:

$$\epsilon \approx \frac{\omega_0^2}{a^2}$$

Stress:

$$\sigma_x \approx \frac{E\omega_0^2}{(1-\upsilon)\ a^2}$$

Stretching energy:

$$U_s \propto \frac{Eh\omega_0^4}{a^2}$$

External work:

$$W \propto p\omega_0a^2$$

Minimizing potential energy $\Pi = U_s - W$:

$$\frac{\partial\Pi}{\partial\omega_0} \propto \frac{Eh\omega_0^3}{a^2} - pa^2 = 0$$

$$\omega_0^3 \propto \frac{pa^4}{Eh},\quad \omega_0 \propto p^{1/3}$$

This nonlinear relationship reduces linearity.

If flexure suppression units are introduced at the bottom of MEMS membrane, it can be approximately regarded as introducing a spring constraint with stiffness $k_{fle} \propto \frac{E_fh_f^3}{L^3}$, where E_f, h_f, L are flexure suppression unit's Young's modulus, thickness, and length.

The flexure suppression unit alters the boundary conditions of the membrane deformation: transitioning from a "fixed boundary" to a condition where "displacement and rotation are permitted at the boundary, with the central deformation ω_0 reduced.

The flexure suppression unit's strain energy is:

$$U_{fle} \approx \frac{1}{2} k_{fle} \omega_{edge}^2$$

Where ω_{edge} is the edge deflection at midpoints, proportional to ω_0. Thus:

$$U_{fle} \propto k_{fle} \omega_0^2$$

Total potential energy:

$$\Pi = U_b + U_s + U_{fle} - W$$

$$U_b \propto D\omega_0^2,\ U_s \propto \frac{Eh\omega_0^4}{a^2},\ U_{fle} \propto k_{fle}\omega_0^2,\ W \propto p\omega_0 a^2.$$

Minimizing Π:

$$\frac{\partial \Pi}{\partial \omega_0} \propto \frac{\partial U_b}{\partial \omega_0} + \frac{\partial U_s}{\partial \omega_0} + \frac{\partial U_{fle}}{\partial \omega_0} - \frac{\partial W}{\partial \omega_0} = 0$$

$$\omega_0 \left(\mathrm{D} + k_{fle} + 2\frac{Eh\omega_0^2}{a^2} \right) \propto pa^2$$

Under small deflection conditions ($\omega_0 \ll h$), The stretching term $\frac{Eh\omega_0^2}{a^2}$ is negligible:

$$\omega_0(\mathrm{D} + k_{fle}) \propto pa^2,\ \omega_0 \propto p$$

The linear relationship is maintained, with flexure suppression units increasing effective stiffness.

Under large deflection conditions ($\omega \sim h$), the flexure suppression unit stiffness k_{fle} limits ω_0, lowering $U_s \propto \omega_0^4$, keeping the membrane in the linear regime ($\omega_0 \propto p$) for a wider pressure range, and delaying the onset of nonlinearity ($\omega_0 \propto p^{1/3}$). The flexure suppression units increase boundary flexibility and effective stiffness, reducing center deflection and suppressing the balloon effect, thus extending the linear regime and improving linearity.

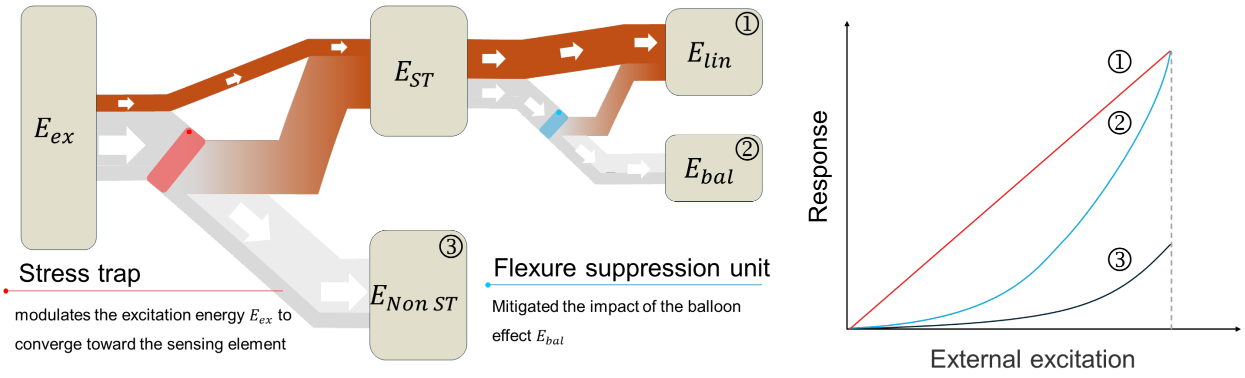

Fig. S10 | The illustration elucidating the mechanisms by which two biomimetic elements enhance sensor performance. E_{ex}: excitation energy, E_{ST}: the energy of stress trap area, $E_{Non\ ST}$: the energy of non stress trap area, E_{lin}: energy in linear regime, E_{bal}: energy in the nonlinear regime induced by the balloon effect.

Table S1 | Material properties of silicon (single crystal, isotropic).

Parameter	Value	Unit
Density	2329	Kg/m^3
Young's modulus	170e9	Pa
Poisson's ratio	0.28	1
Thermal expansion coefficient	2.6e-6	1/K
CPMX	700	J/(kg*K)
thermal conductivity	130	W/(m*K)

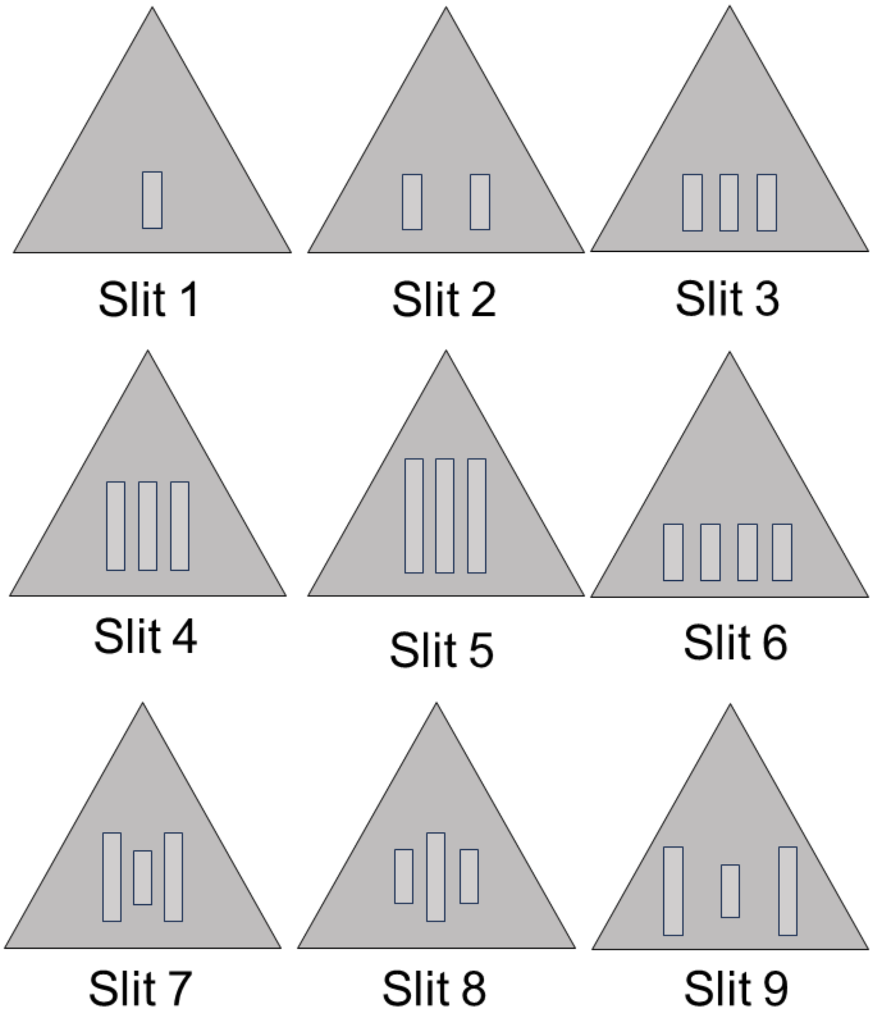

Fig. S11 | Nine bioinspired silt structures (stress traps) for finite element optimization analysis. The study investigates the impact of the number, shape, and distribution pattern of slits on the stress concentration in the surrounding region, under the constraints of the resistor arrangement required for the Wheatstone bridge.

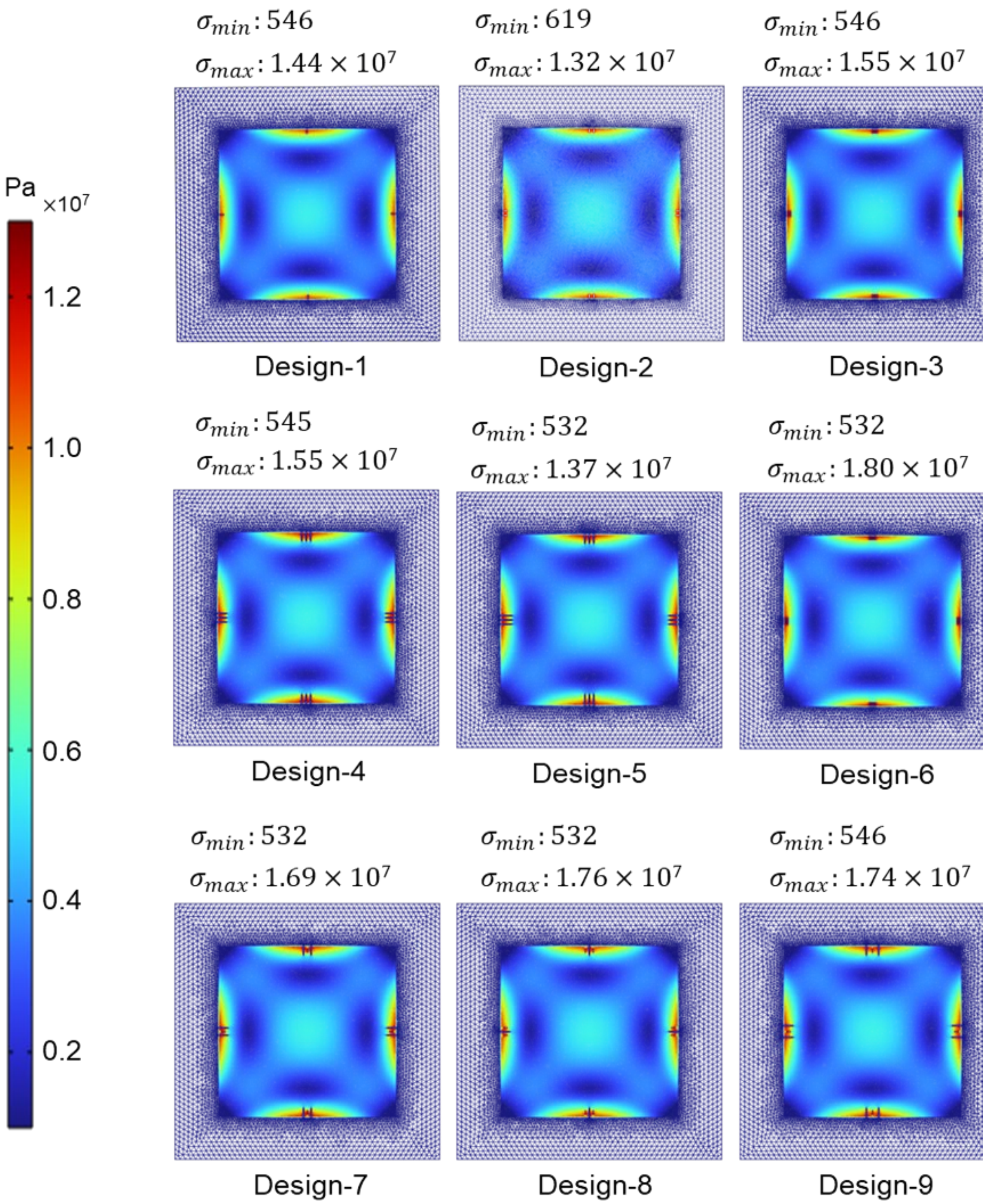

Fig. S12 | Stress analysis for Design-1 to Design-9. Numerical snapshots extracted from linear FE analysis (with first mode-imposed imperfection) at an imposed constant pressure P=1 kPa.

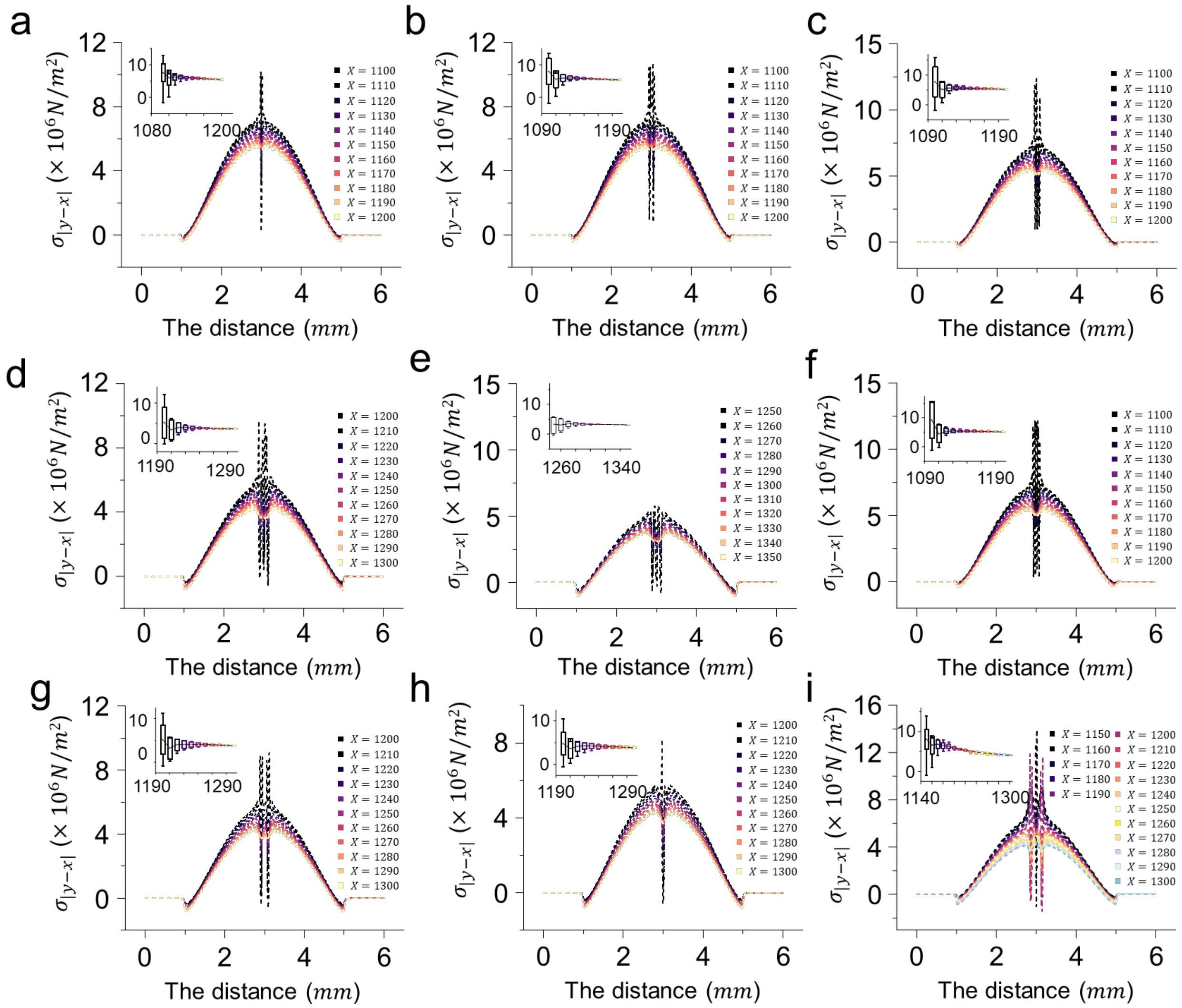

Fig. S13 | The design of R_d' position for Design-1 to Design-9. Stress value extracted from linear FE analysis at an imposed constant pressure P=1 kPa. The difference $\sigma_{|y-x|}$ of the second Piola–Kirchhoff stress component σ_x and σ_y from 0 to 6000 are shown here to determine the most suitable position for R_d settings for each structure of Design-1 to Design-9 indicated above each figure. **a**, Design-1. **b**, Design-2. **c**, Design-3. **d**, Design-4. **e**, Design-5. **f**, Design-6. **g**, Design-7. **h**, Design-8. **i**, Design-9.

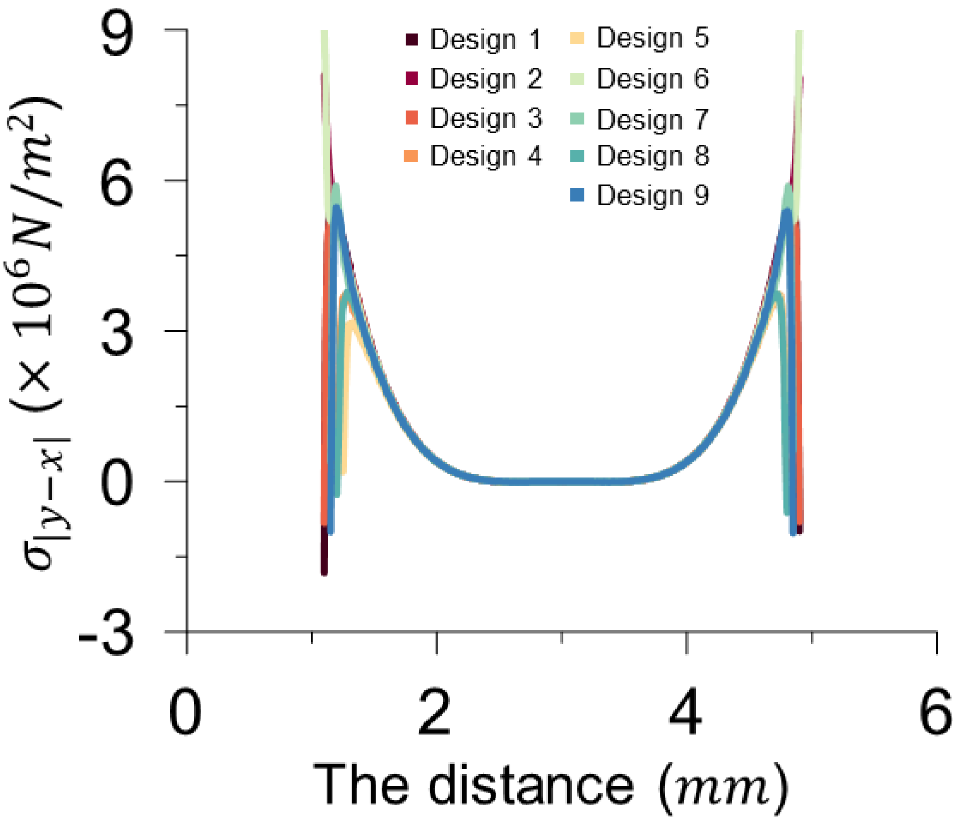

Fig. S14 | The design of R_i' position for Design-1 to Design-9. The difference $\sigma_{|y-x|}$ of the second *Piola–Kirchhoff* stress component σ_x and σ_y from y = 3000 are shown here to determine the most suitable position for R_i settings for each structure of Design-1 to Design-9.

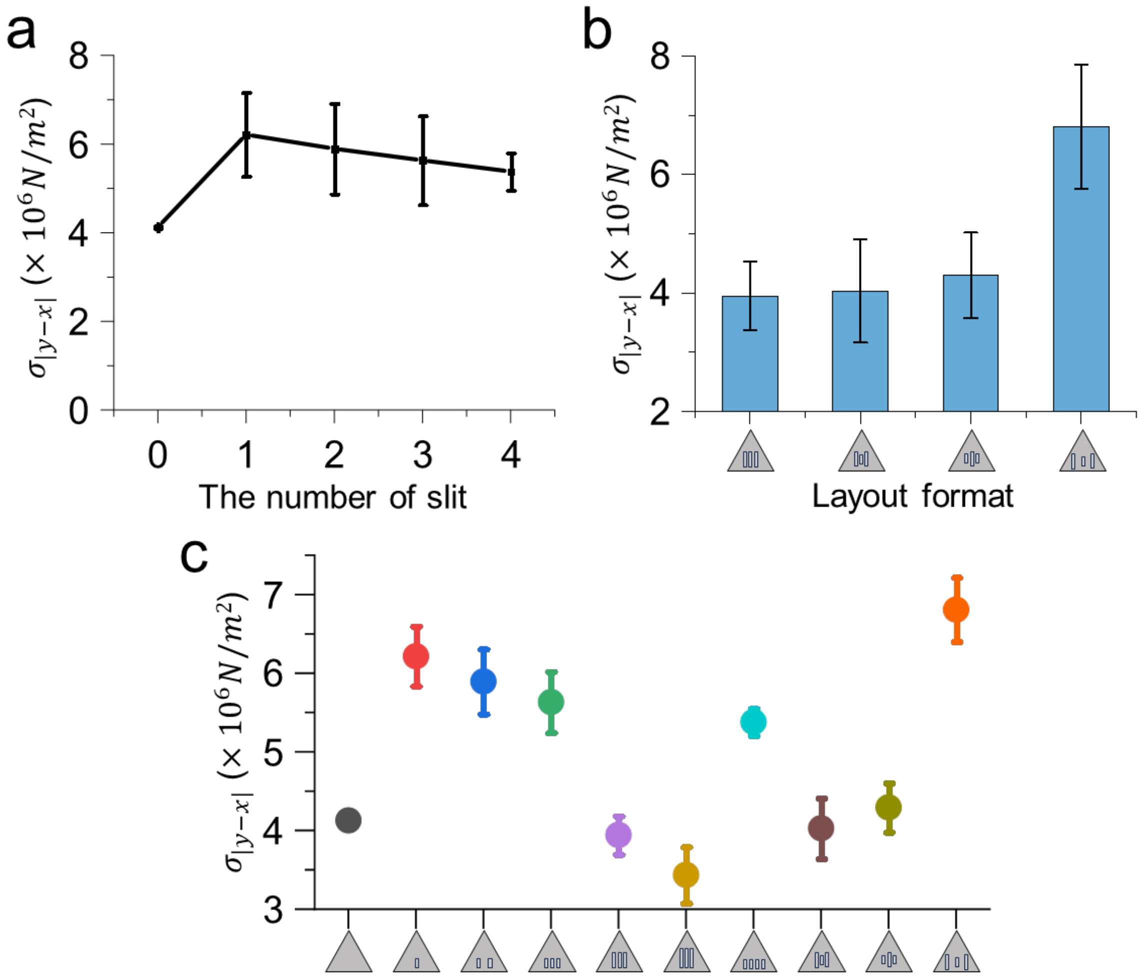

Fig. S15 | The optimization of the stress trap structure to enhance the stress concentration around $\boldsymbol{R_i}$. (a) Optimization of the number of slit. (b) Optimization of the layout format. (c) The average stress induced around $\boldsymbol{R_i}$ by nine different stress trap structures is used to determine which design scheme to adopt for $\boldsymbol{R_i}$.

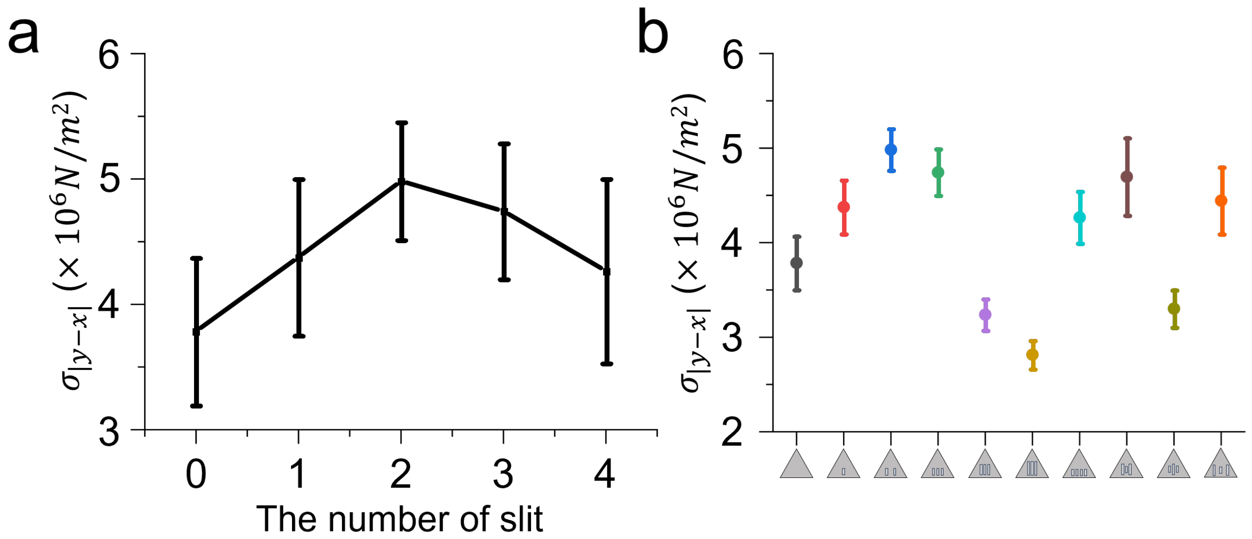

Fig. S16 | The optimization of the stress trap structure to enhance the stress concentration around R_d. (a) Optimization of the number of slits. (b) The average stress induced around R_d by nine different stress trap structures is used to determine which design scheme to adopt for R_d.

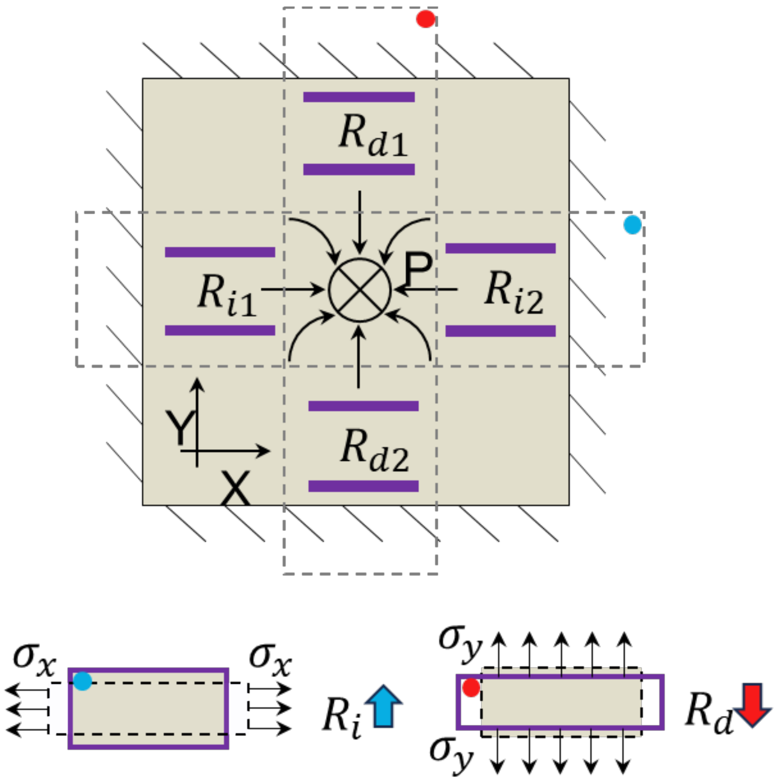

Fig. S17 | Schematic diagram of the forces on the chip. The resistance value of R_i increases due to lateral stretching. Conversely, the resistance value of R_d decreases due to longitudinal stretching.

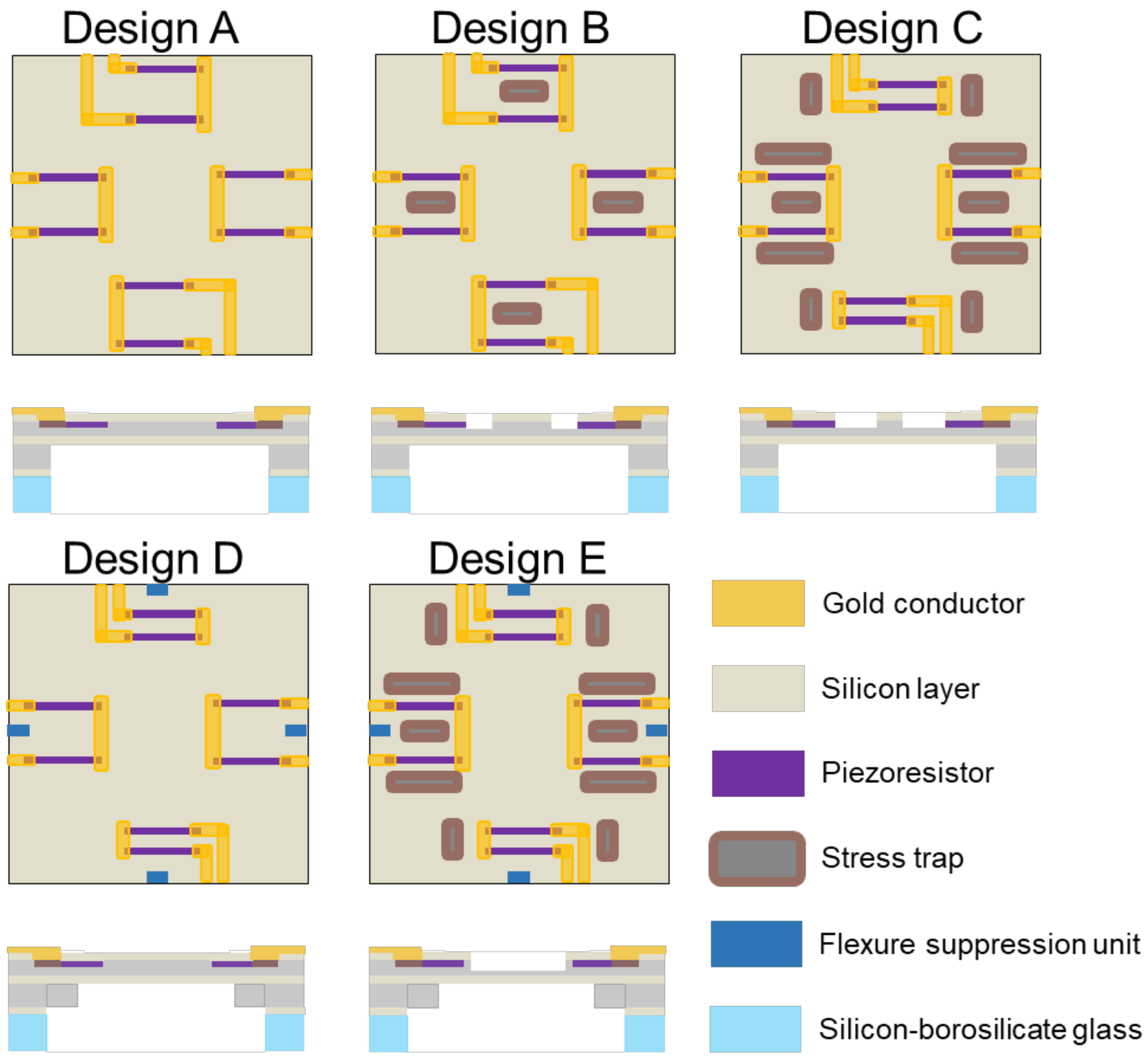

Fig. S18 | Top view and sectional view Schematics of Designs A-E.

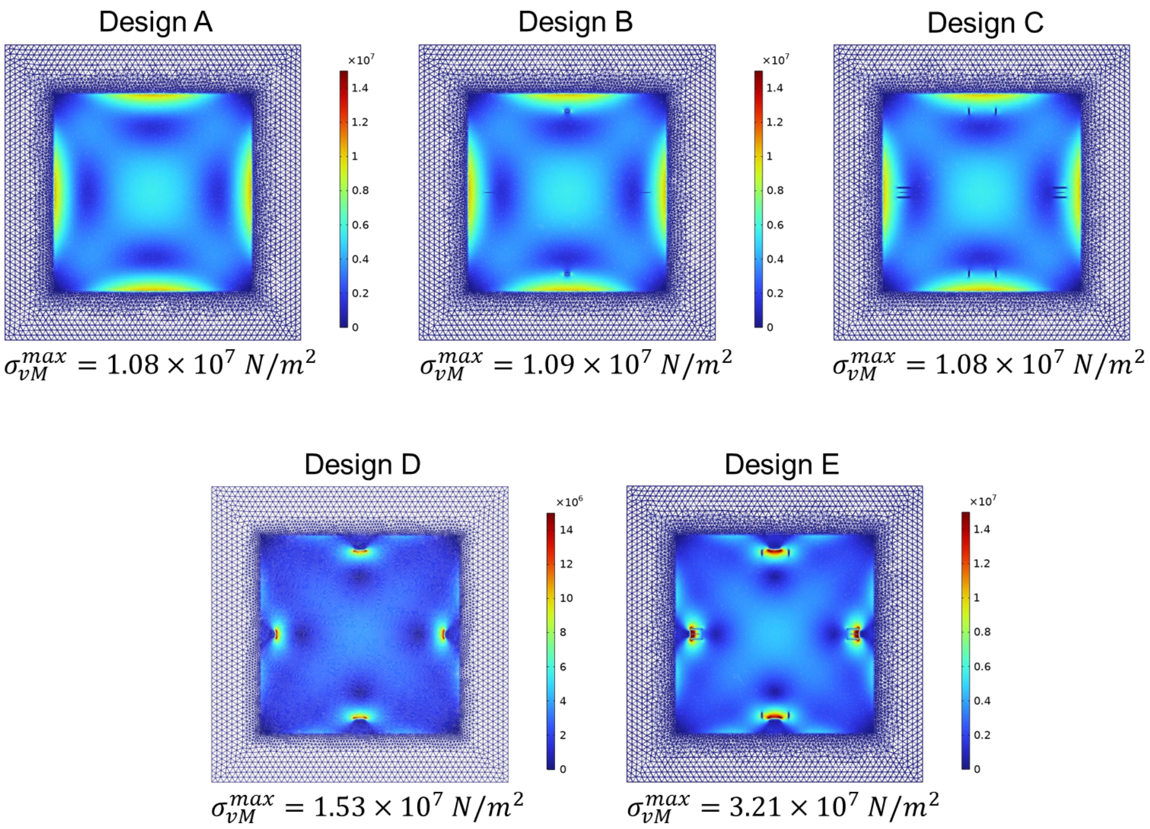

Fig. S19 | Stress analysis for Designs A-E. Stress cloud map (wireframe) extracted from linear FE analysis at an imposed constant pressure $P = 1\ kPa$. The color indicates the *von Mises* stress σ_{vM} with the maximum value for each *von Mises* indicated above each figure.

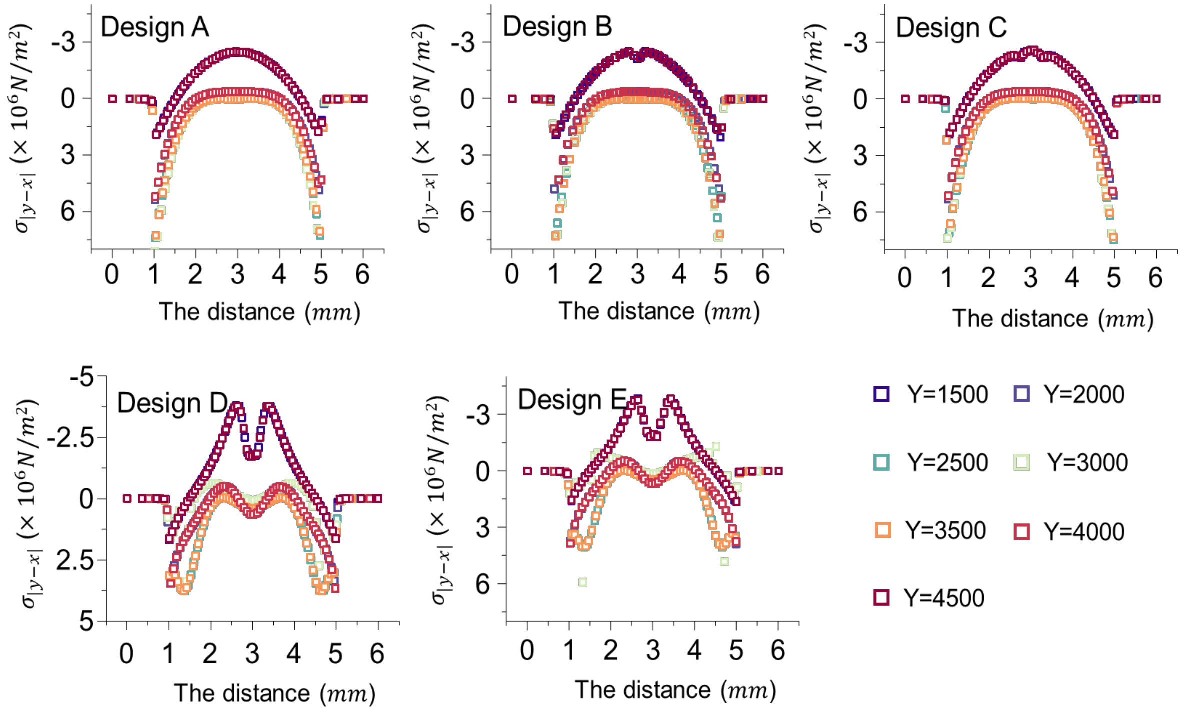

Fig. S20 | **Response of Designs A-E from $y = 1500$ to $y = 4500$.** Stress value extracted from linear FE analysis at an imposed constant pressure $P = 1\ kPa$. The difference $\sigma_{|y-x|}$ of the second Piola–Kirchhoff stress component σ_x and σ_y from 1500 to 4500 are shown here for each structure of Designs A-E indicated above each figure. When y=3000, the stress concentration area gathers on both sides and exhibits a sharp mutation. As the stress line approaches the edge, the stress value in the central section increases. Therefore, both R_i and R_d need to be distributed on the four sides of the membrane to achieve greater stress concentration.

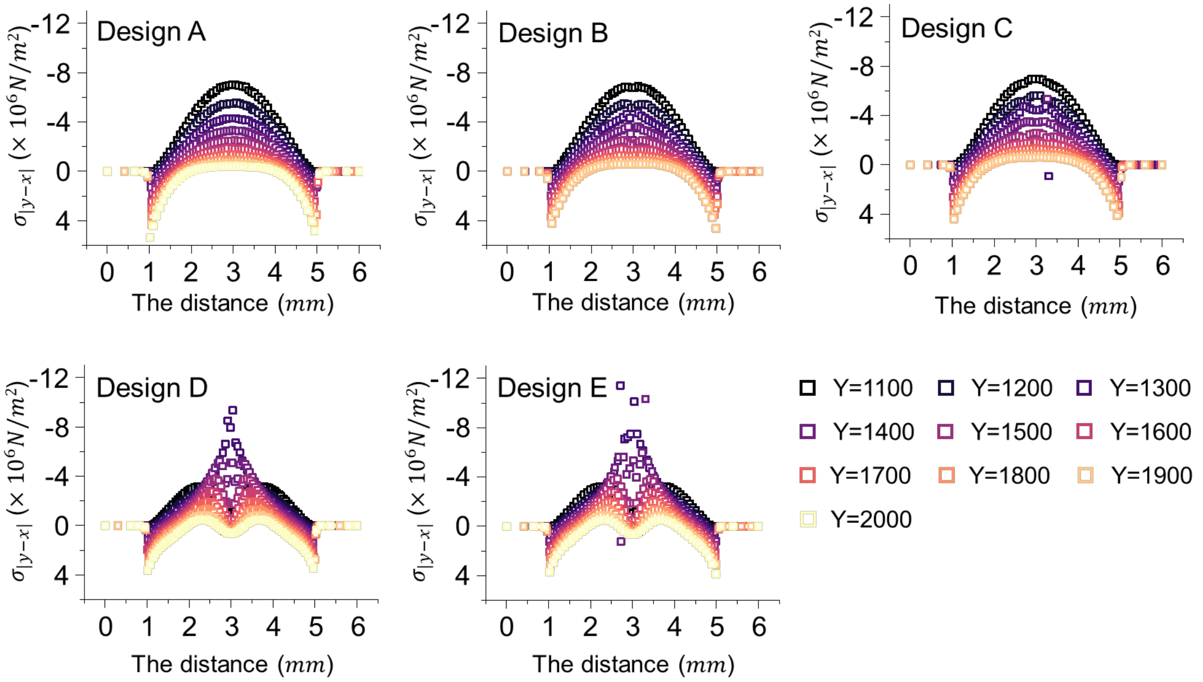

Fig. S21 | Optimization of the distribution position of R_d from $y = 1100$ to $y = 2000$. The $\sigma_{|y-x|}$ of Designs A-E are extracted from linear FE analysis. For Designs A-C, it is most reasonable to place the piezoresistor near the y = 1100 region, as this area experiences the highest stress. In contrast, for Designs D and E, placing the piezoresistor near y = 1300 is the most optimal, given the stress distribution in these regions.

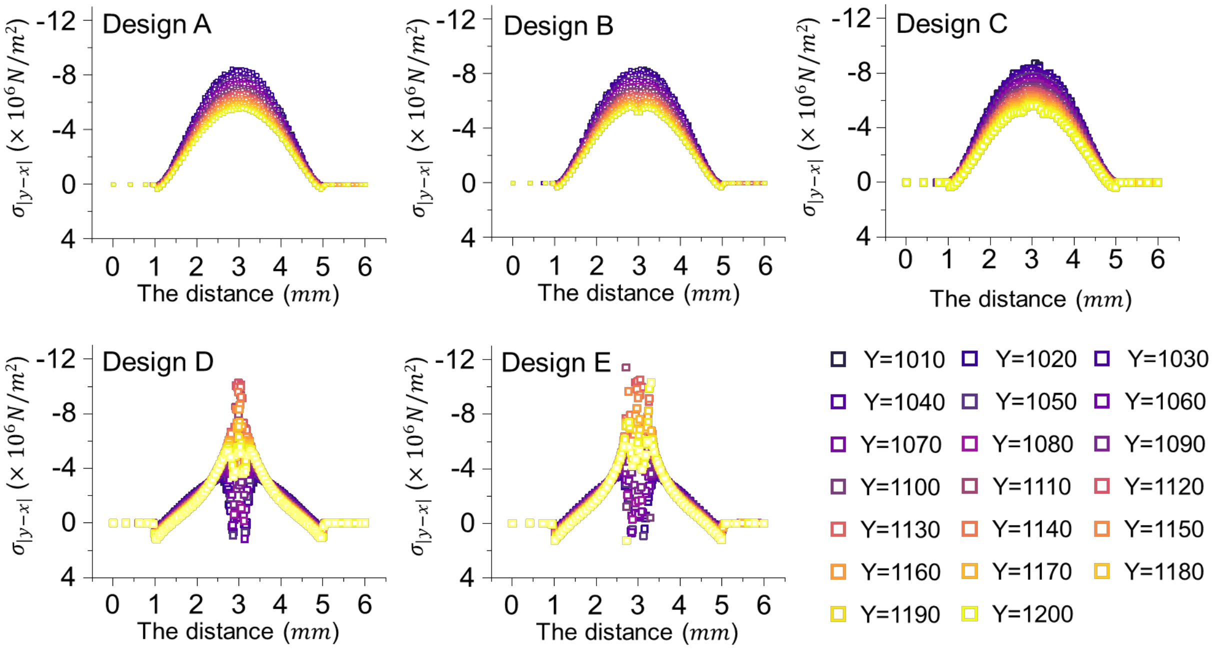

Fig. S22 | Optimization of the distribution position of R_d for Designs A-E. The $\sigma_{|y-x|}$ for y=1010 to y=1200 with 10um gaps between each curve are extracted from linear FE analysis.

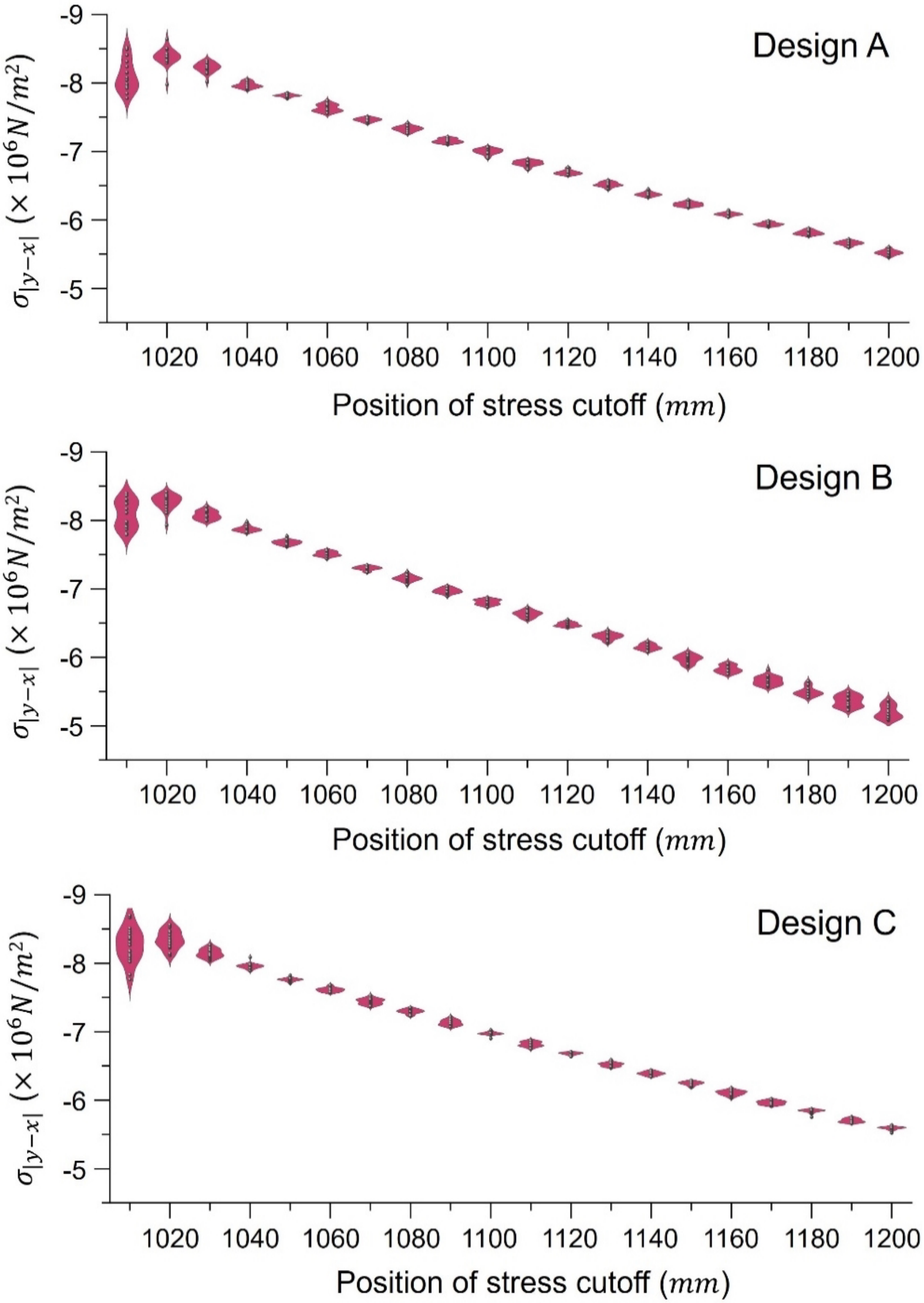

Fig. S23 | Distribution of stress values in Designs A-C. Based on the principle of seeking high values and uniformity of stress distribution in the layout area of piezoresistors, for Design A, Design B and Design C, y=1030 is chosen as R_d's layout line.

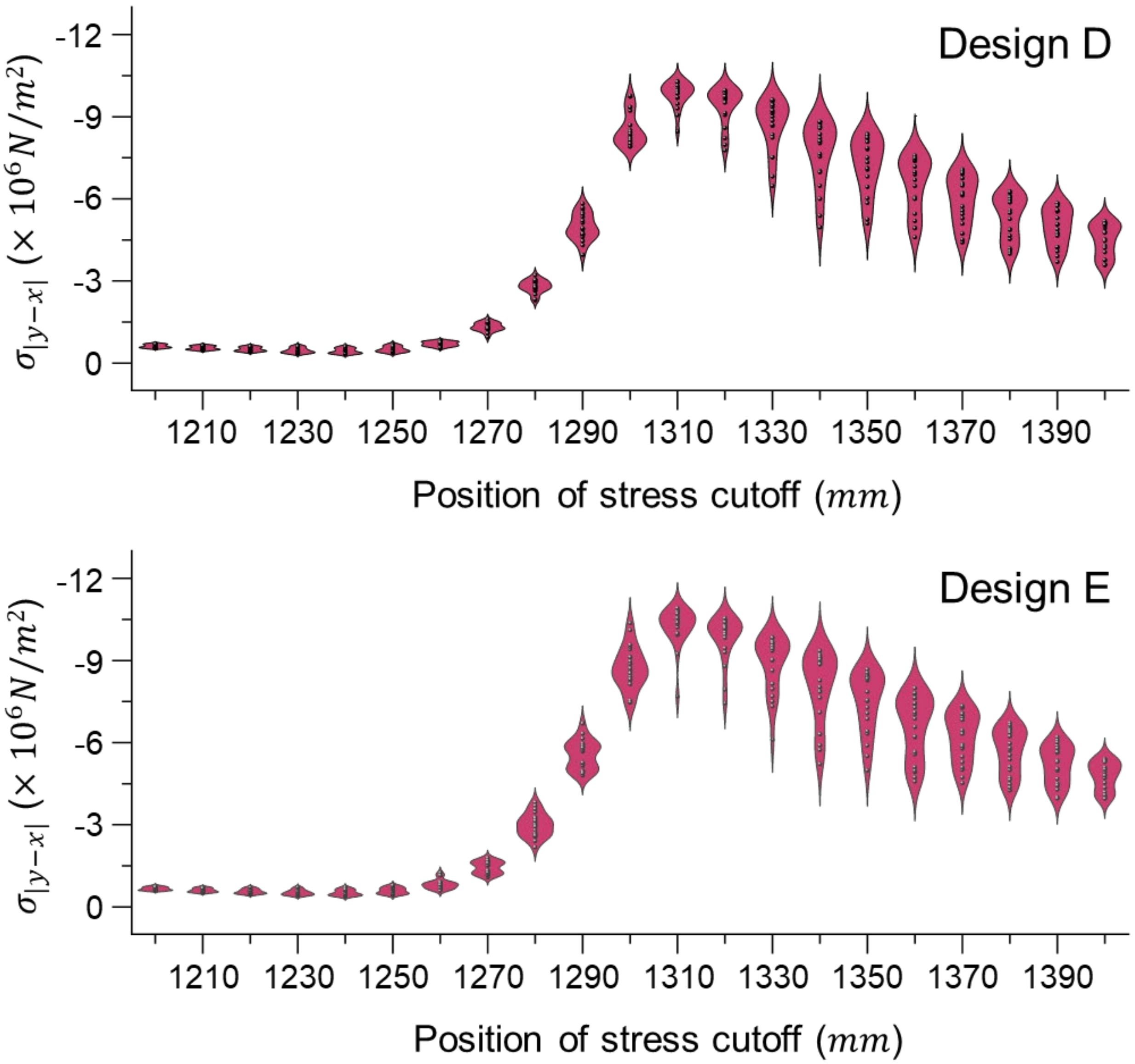

Fig. S24 | Distribution of stress values for $y = 1200$ to $y = 1400$ from $x = 2900 \sim 3100$ in Design D and Design E. The distribution of stress values for $y = 1310$ from $x = 2900 \sim 3100$ shows the highest values and least dispersion.

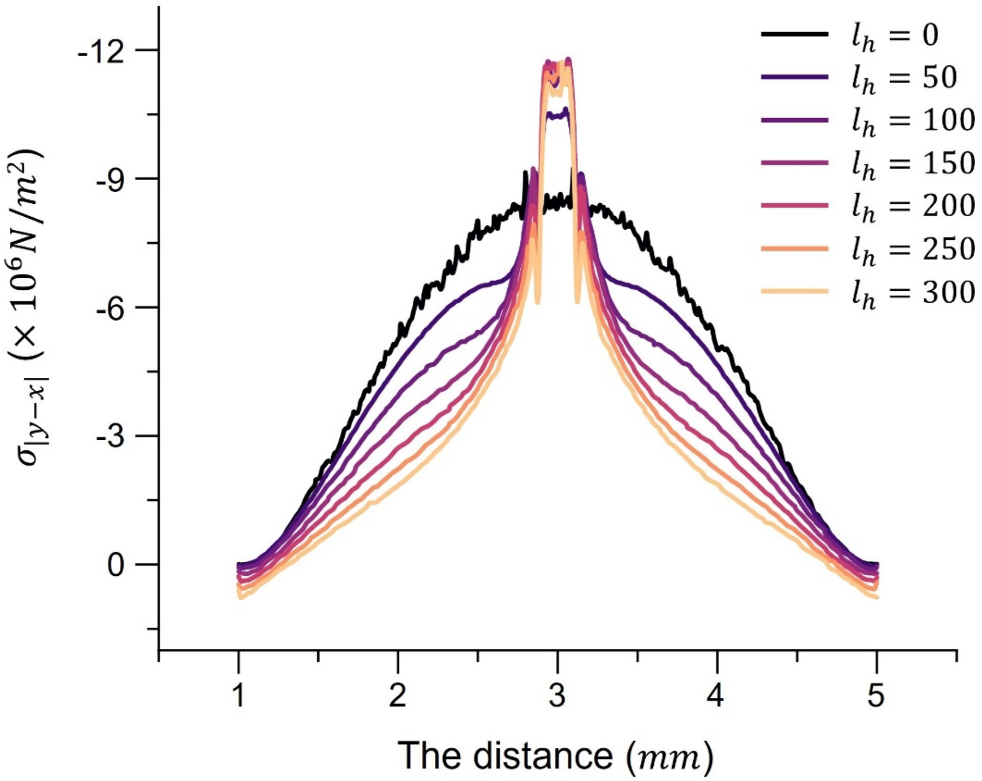

Fig. S25 | Distribution of stress values for different sizes of flexure suppression unit from $l_h = 0\sim300\ mm$.

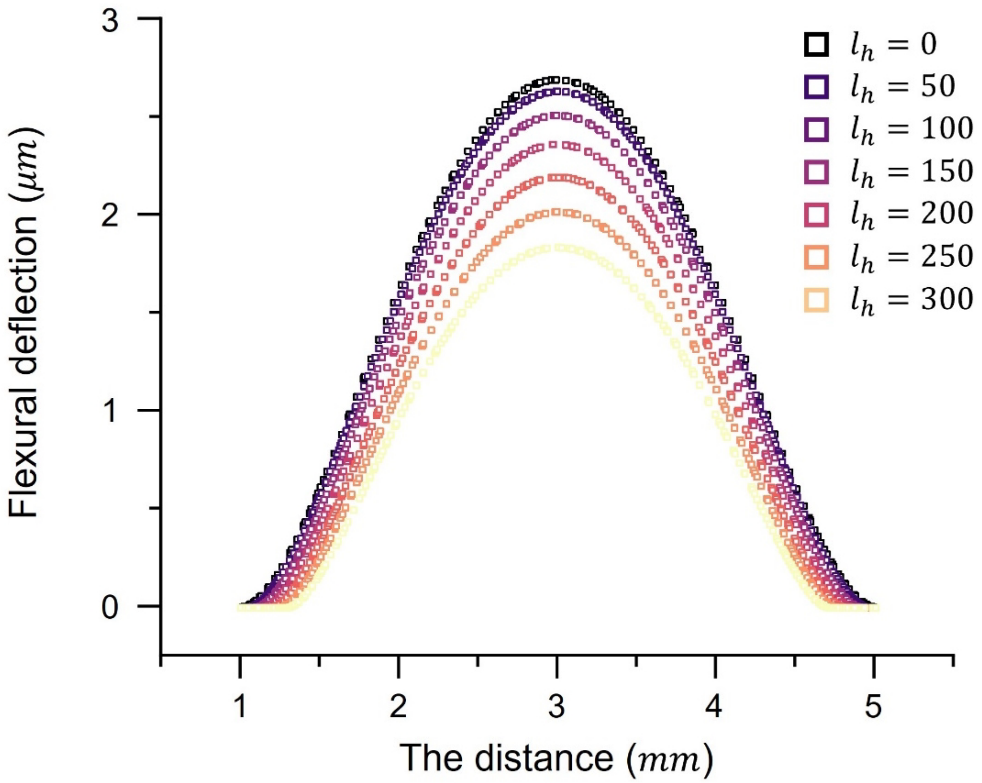

Fig. S26 | Distribution of flexural deflection. The influence of flexure suppression unit on the stress distribution of membrane with stress traps set at y=1310 has been further studied for different sizes of flexure suppression unit from $l_h = 0{\sim}300\ mm$.

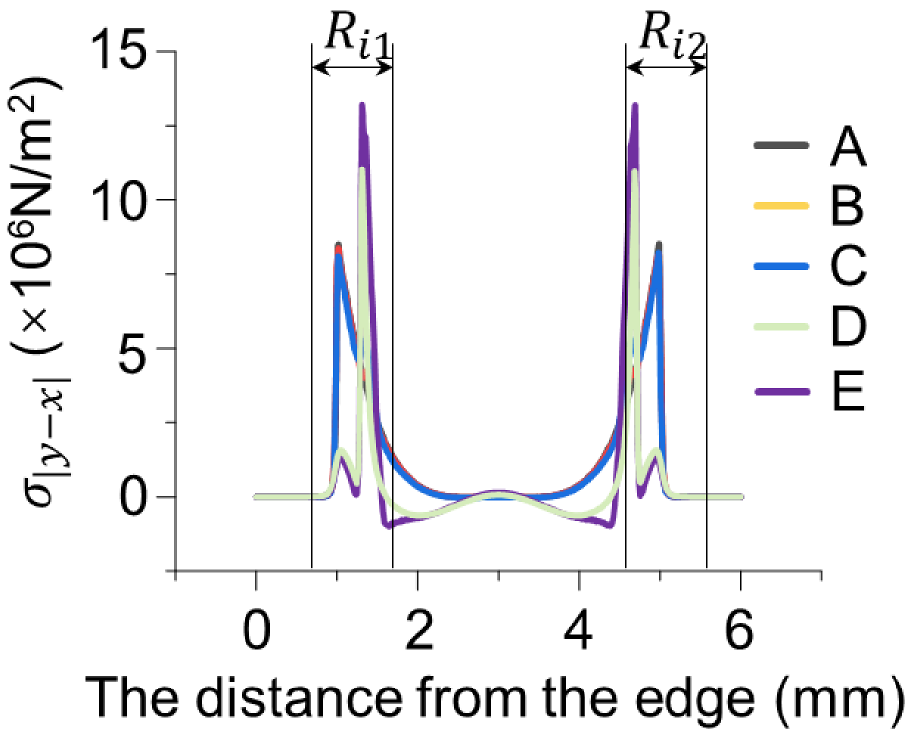

Fig. S27 | The $\sigma_{|y-x|}$ of Designs A-E when $y = 3000\ \mu m$. The stress distribution values along this line are employed to study the spatial distribution of the piezoresistors R_i.

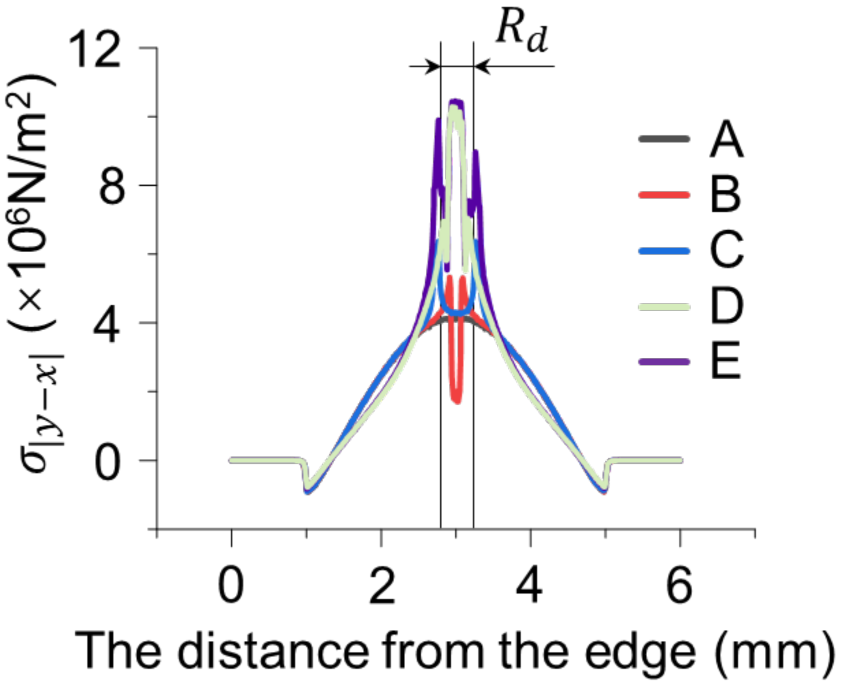

Fig. S28 | The $\sigma_{|y-x|}$ of Designs A-C when $y = 1030\ \mu m$, and Designs D-E when $y = 1310\ \mu m$. The stress distribution values along this line are employed to study the spatial distribution of the piezoresistors R_d.

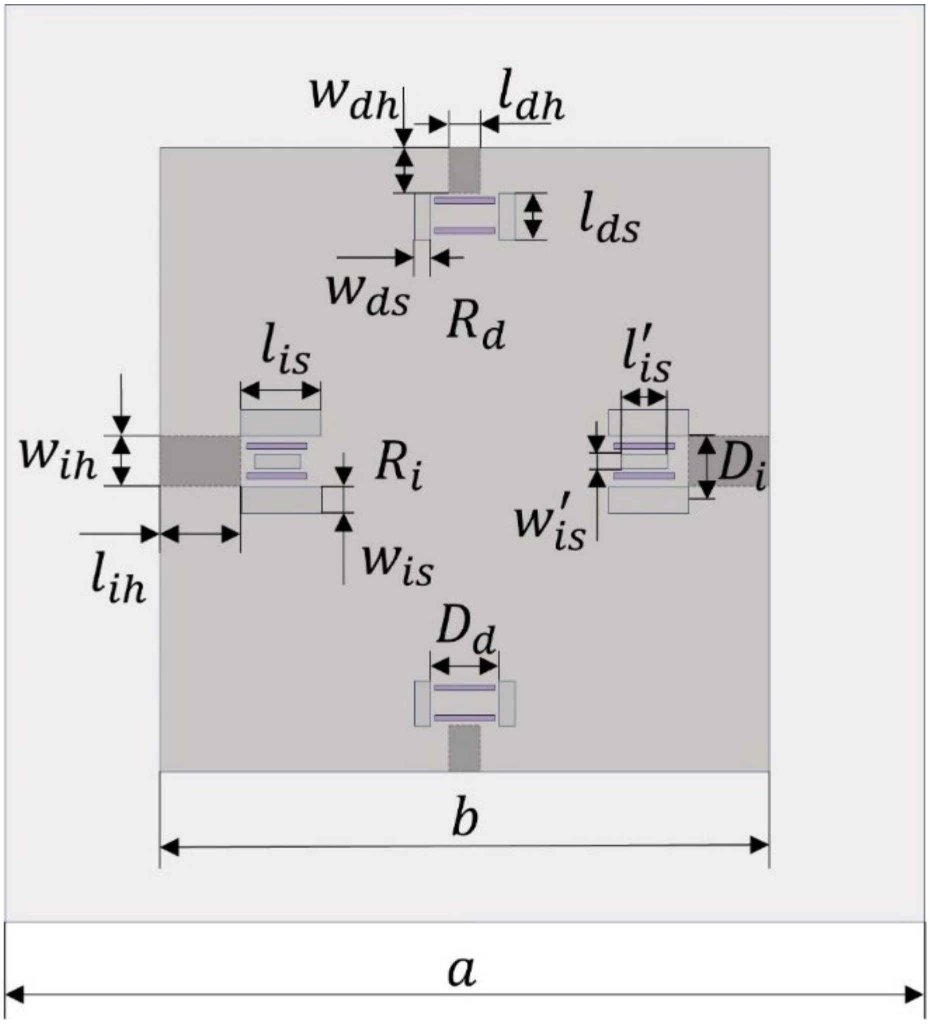

Fig. S29 | Schematic diagram of the optimized sensor structures. For readers to observe more clearly, some feature structures have been appropriately enlarged, and the relevant true sizes are indicated in Table S2.

Table S2 | List of BPPS size values.

Nomenclature	value [μm]	Nomenclature	Design value [μm]
a	6000	w_{is}	50
b	4000	l_{is}	250
w_{ih}	160	w'_{is}	25
l_{ih}	300	l'_{is}	140
w_{dh}	300	w_{ds}	50
l_{dh}	250	l_{ds}	100
D_d	470	D_i	145

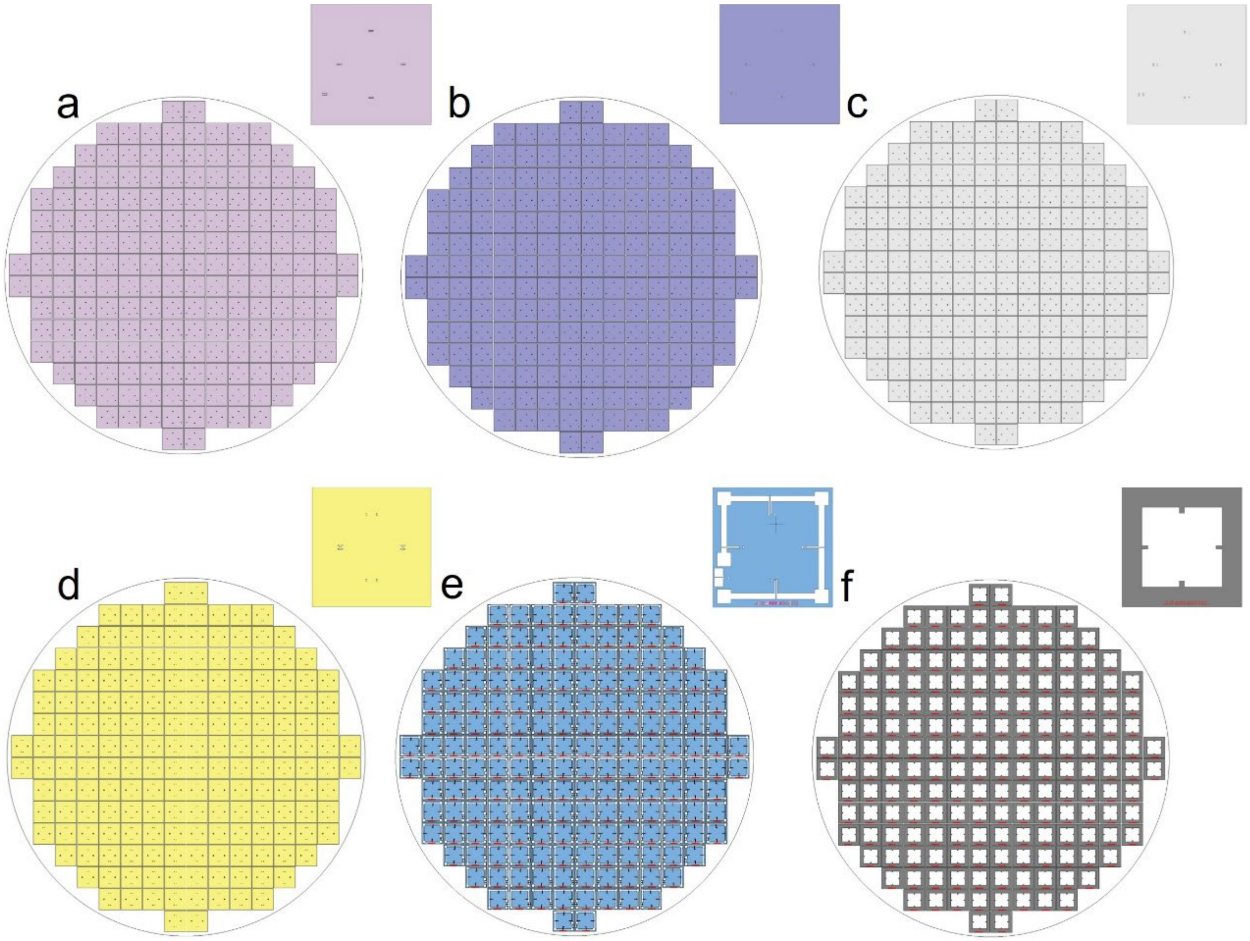

Fig. S30 | The masks used in the fabrication process of BPPS. a, Lightly doped; **b**, Heavily doped; **c**, Exposing the bond pad; **d**, Electrode deposition; **e**, Gold wire deposition; **f**, Bottom silicon etching.

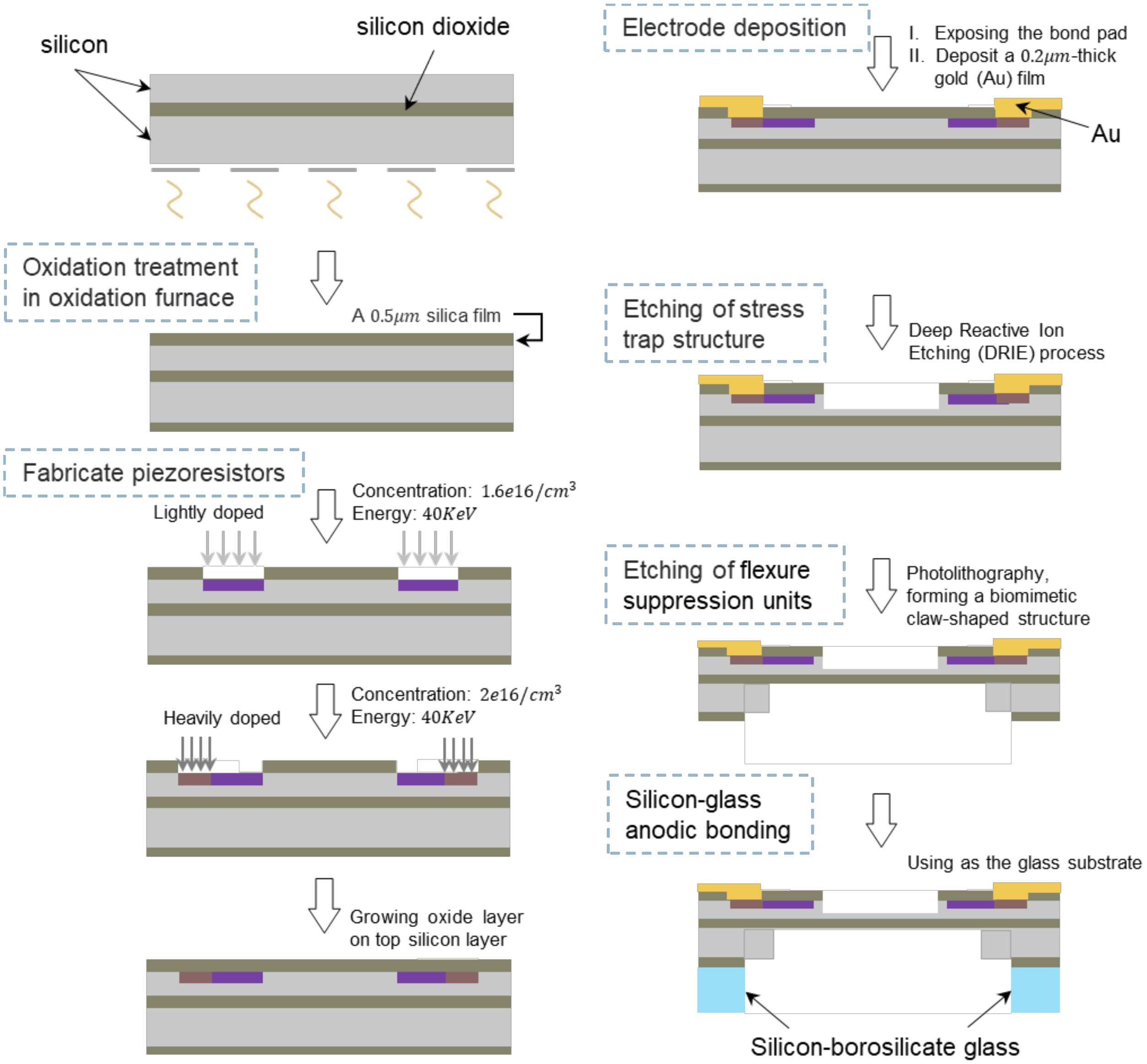

Fig. S31 | Schematics for the fabrication process of BPPS.

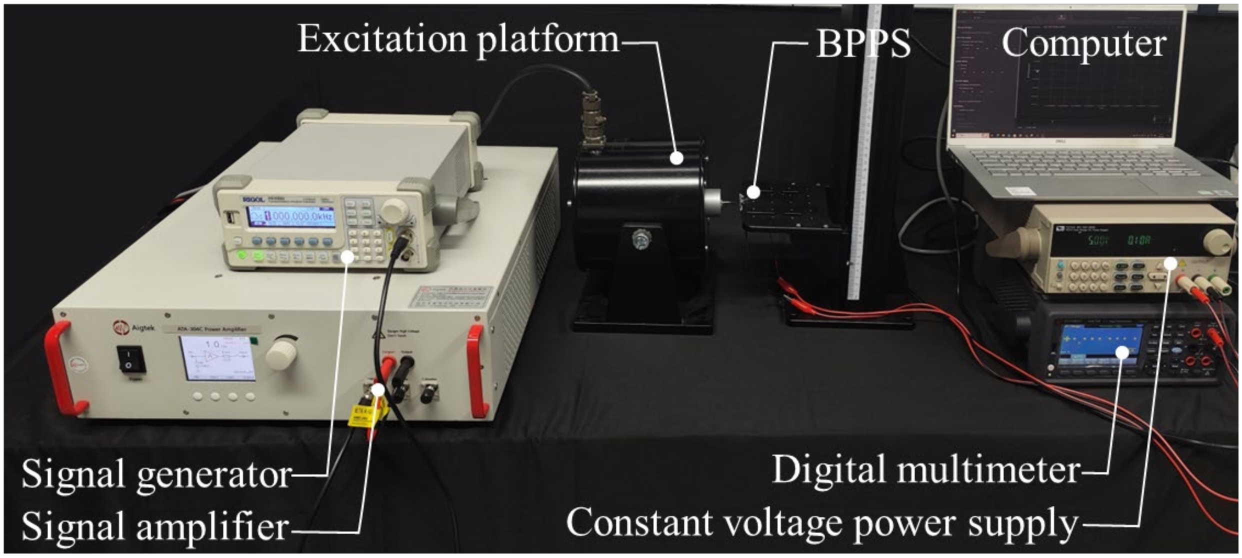

Fig. S32 | Custom-designed pressure stimulation test platform.

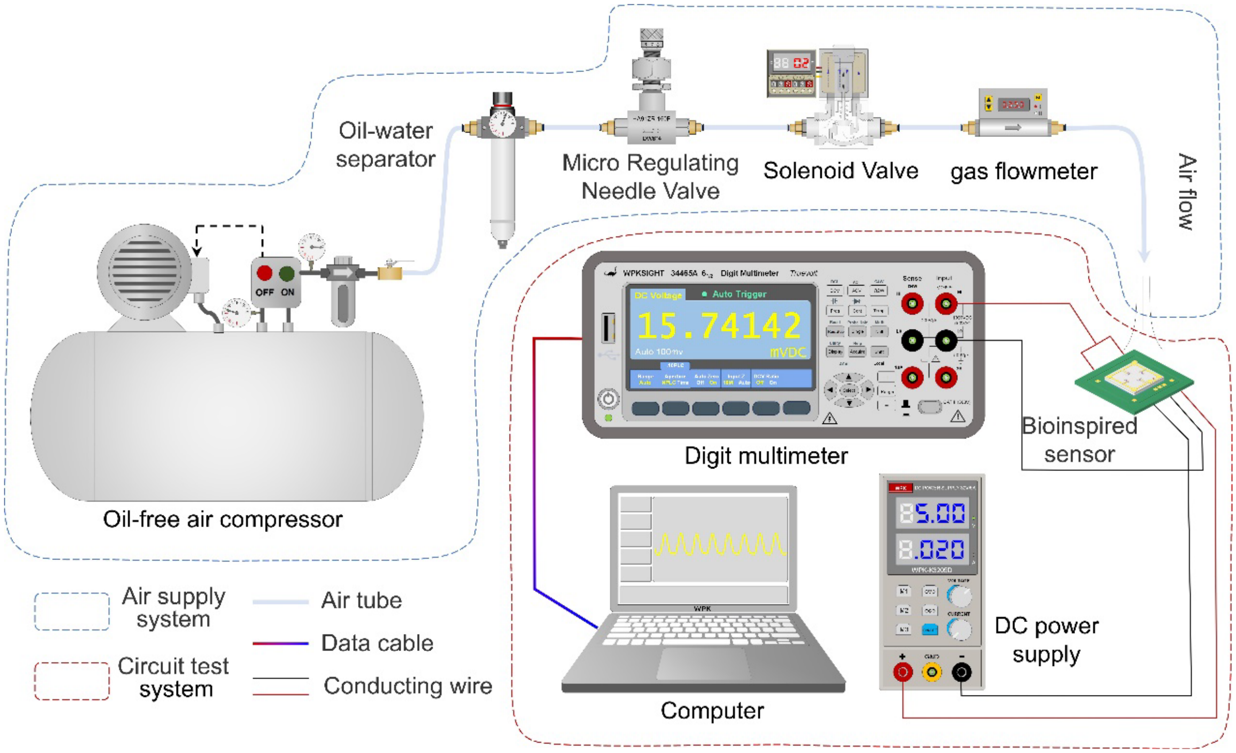

Fig. S33 | Airflow stimulation testing system. It comprises the controllable airflow stimulation (air supply system) and real-time recording of BPPS output (circuit test system) respectively encircled by blue dashed and red dashed lines in the diagram.

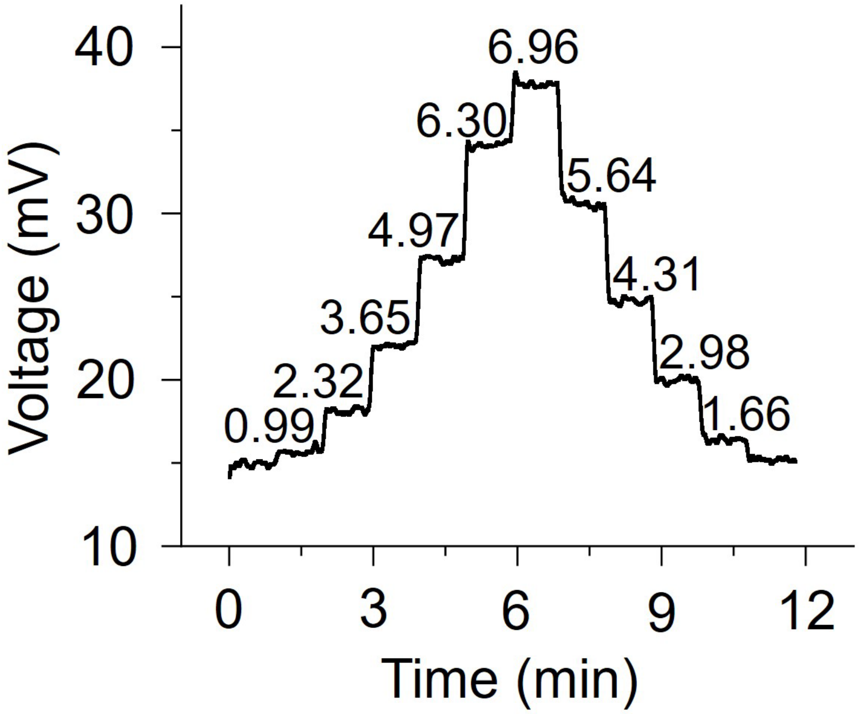

Fig. S34 | Response to the airflow. Real-time monitoring of BPPS output voltage changes as wind speed was stepped up and down.

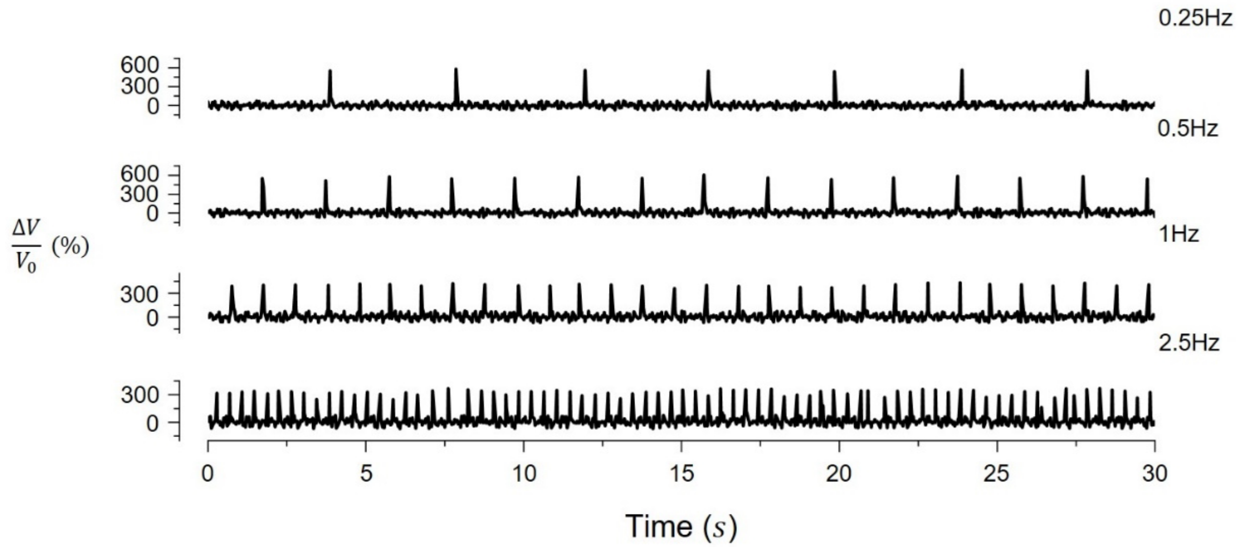

Fig. S35 | Performance testing at different frequencies with the same amplitude. In the case of pulse airflows with the same amplitude but different frequencies, the sensor produces a noticeable response to each stimulus. However, the magnitude of the response decreases with increasing frequency, which is attributed to the increased response time of the air supply system caused by the complex piping of the self-built testing system.

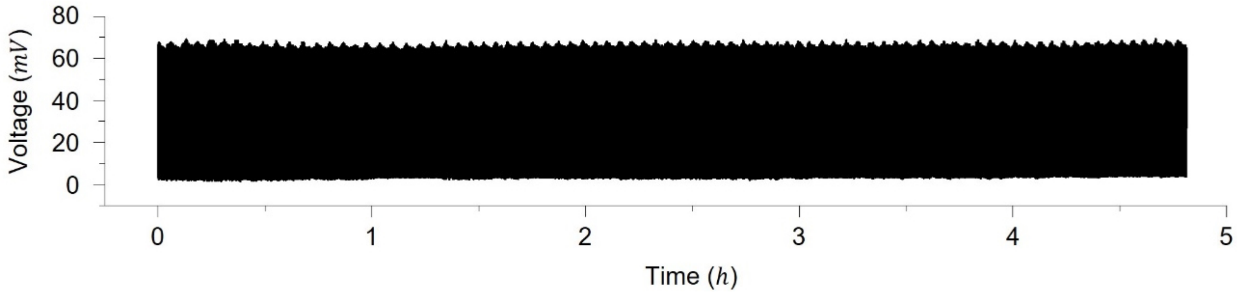

Fig. S36 | Fatigue testing of response to airflow. Continuous monitoring of periodic pulse airflow (T = 6s) is achieved steadily by BPPS for approximately 5 h.

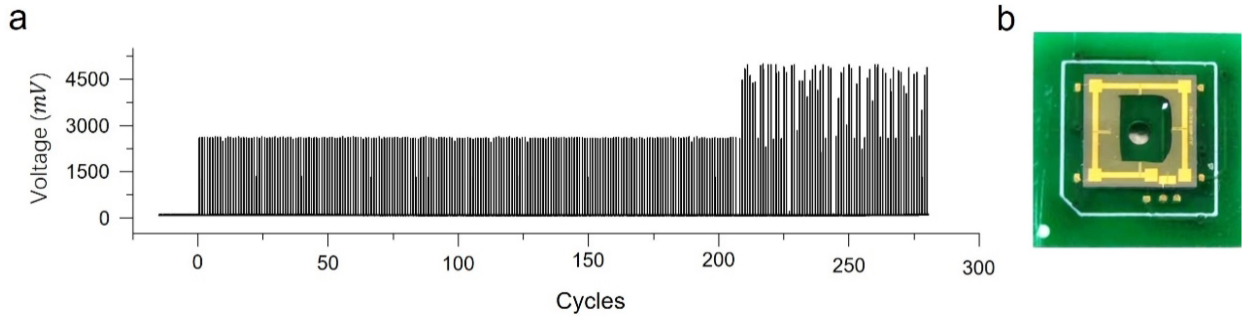

Fig. S37 | Destructive testing of BPPS using air blast to study its reliability. a, Response curve. The detection limit of BPPS was validated using a strong airflow of 28.4 m/s. After undergoing 206 strong pulse impacts, BPPS suffered from failure and damage. **b**, The physical image of BPPS after the destructive test.

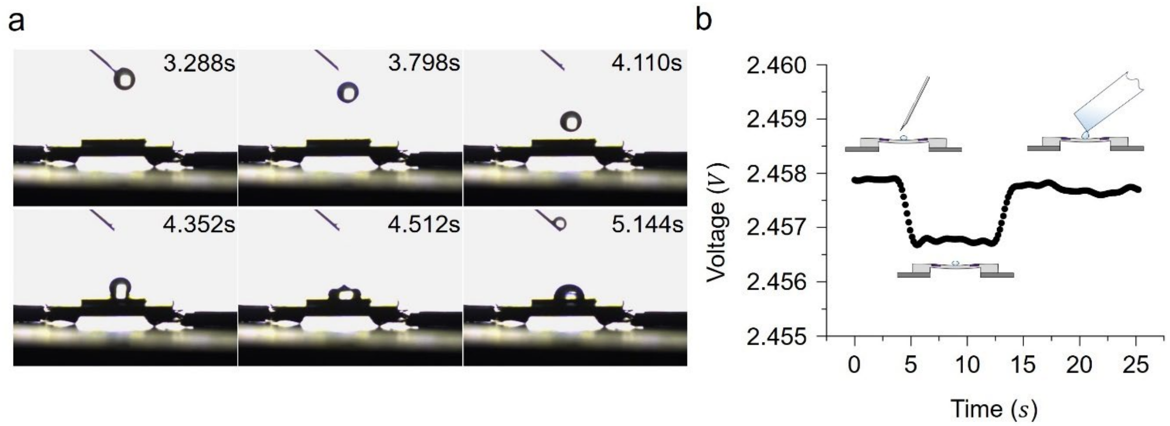

Fig. S38 | Water drop test demonstrating the sensor's ability to respond to light pressure. a, The snapshots of droplets bouncing on BPPS. **b,** Output curve of droplet testing process.

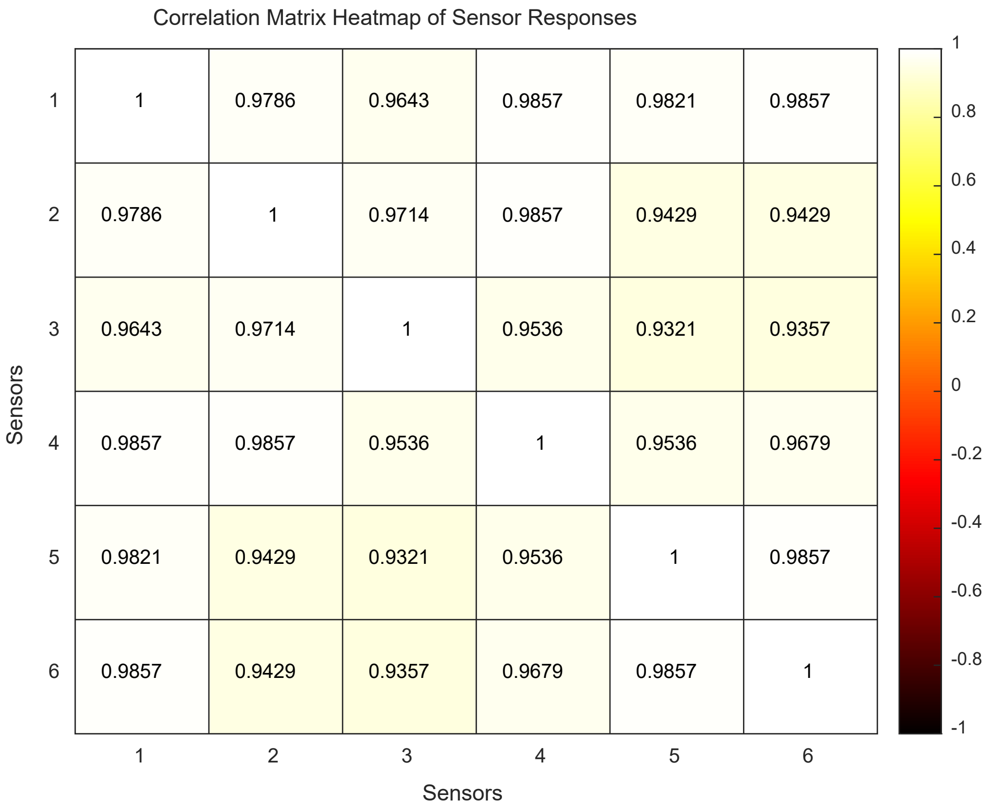

Fig. S39 | Correlation matrix heatmap of sensor responses. The Spearman correlation coefficient is used to evaluate the response consistency of six sensors, with values close to 1 indicating a high degree of response consistency among the sensors.

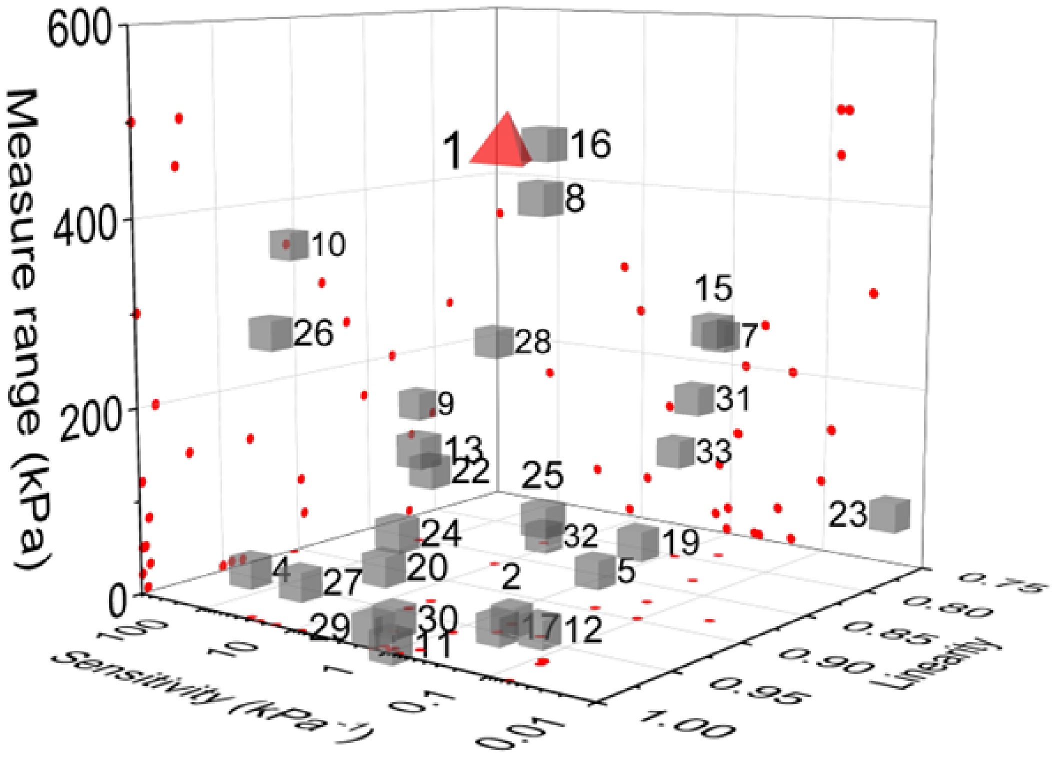

Fig. S40 | **Comparison of BPPS with previously reported pressure sensors in three key performance parameters: sensitivity (preferred to be higher), and linearity (needs to approach 1), measure range (preferred to be wider).**

Table S3 | **Comparison of our sensor with previously reported pressure sensors.**

No	Research	Sensitivity (kPa^{-1})	Linearity	Measure range (kPa)
	This work	0.06556	0.99934	500
Ref 1	*Nat. Electron.* **7**, 168–179 (2024)	0.5395	0.93704	6
Ref 2	*Nat. Commun.* **15**, 5596 (2024)	0.3071	0.89545	40
Ref 3	*Nat. Commun.* **14**, 2907 (2023)	32.94	0.997	50
Ref 4	*Nat. Commun.* **13***,* 1743 (*2022*)	0.44274	0.78785	250
Ref 5	*Nat. Commu.* **13**, 1317 (2022)	0.07757	0.97425	450
Ref 6	*Nat. Commun.* **12**, 1776 (2021)	123.8258	0.85258	160
Ref 7	*Nat. Commun.* **11**, 209 (2020)	505.14055	0.90501	350
Ref 8	*Nat. Commun.* **5**, 3132 (2014)	1.08417	0.99615	5
Ref 9	*Nat. Mater.* **9**, 859-864 (2010)	0.2195	0.94405	7
Ref 10	*Microsyst. Nanoeng*. **9**, 5 (2023)	0.705	0.989	200
Ref 11	*Nano-Micro Lett*. **16**, 267 (2024)	0.03456	0.88133	300
Ref 12	*Nano Energy* **121**, 109252 (2024)	0.07967	0.9711	500
Ref 13	*Adv. Mater*. **36**, 2403880 (2024)	0.48328	0.94979	5
Ref 14	*Adv. Mater*. 2406235 (2024)	0.10828	0.89728	80
Ref 15	*Adv. Mater*. **34**, 2200517 (2022)	1.3359	0.99442	80
Ref 16	*Adv. Funct. Mater*. **34**, 2314479 (2024)	4.83523	0.93075	140
Ref 17	*Adv. Funct. Mater*. 2403268 (2024)	0.00394	0.81786	100
Ref 18	*Adv. Funct. Mater*. **34**, 2312370 (2024)	0.8335	0.99814	120
Ref 19	*Adv. Funct. Mater*. 2403788 (2024)	0.0872	0.96921	140
Ref 20	*Adv. Funct. Mater*. 2406762 (2024)	16.24	0.999	300
Ref 21	*Adv. Funct. Mater*. **34**, 2401415 (2024)	8.56644	0.99882	50
Ref 22	*Adv. Fiber Mater*. **6**, 414–429 (2024)	10.49488	0.86447	250
Ref 23	*Adv. Sci.* **10**, 2206807 (2023)	1.448	0.9991	21
Ref 24	*Small* **14**, 1800819 (2018)	1.05789	0.99437	30
Ref 25	*Nano Energy* **126**, 109701 (2024)	0.22316	0.83181	200
Ref 26	*Adv. Compos. Hybrid Mater.* **7**, 24 (2024)	13.69389	0.81994	8
Ref 27	*npj Flex. Electron*. **6**, 92 (2022).	0.85569	0.80171	120

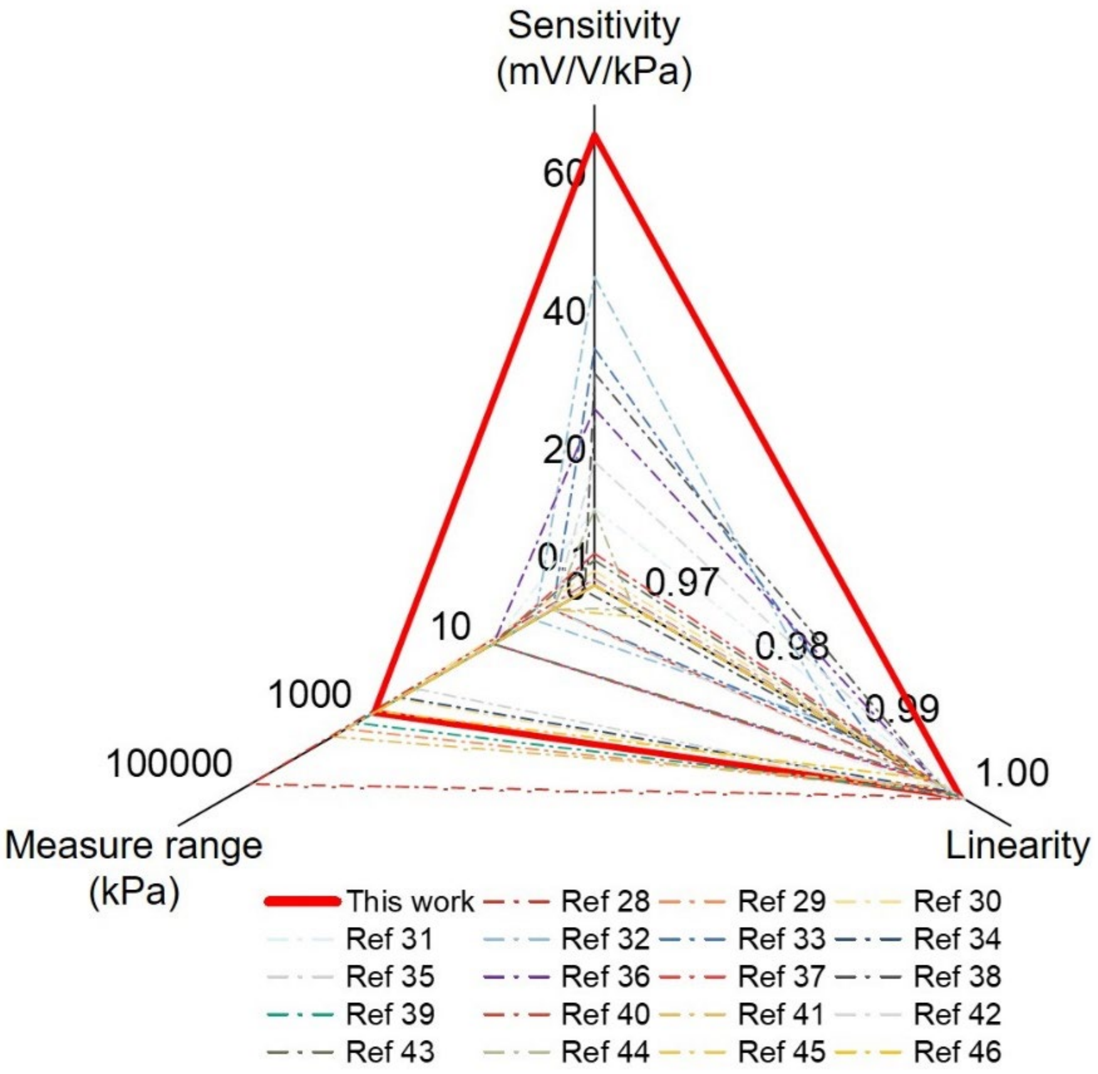

Fig. S41 | Comparison of BPPS with previously reported MEMS piezoresistive pressure sensors.

Table S4 | Comparison of our sensor with previously reported MEMS piezoresistive pressure sensors.

No	Research	Sensitivity (mV/V/kPa)	Linearity	Measure range (kPa)
	This work	65.56	0.99934	500
Ref 28	*IEEE Sens. J.* **23**, 1052-1059 (2023)	0.943	0.99937	60000
Ref 29	*Sensor. Actuat. A-phys.* **364**, 114834 (2023)	0.0093	0.999	1500
Ref 30	*IEEE MEMS.* 901-904 (2023)	2.09	0.9995	200
Ref 31	*Phys. Scr.* **96**, 065705 (2021)	11.2	0.9989	5
Ref 32	*IEEE Sens. J.* **21**, 4357-4364 (2021)	44.9	0.988	1
Ref 33	*IEEE Sens. J.* **20**, 7646-7652 (2020)	34.5	0.9919	0.5
Ref 34	*Sensors* **20**, 337 (2020)	0.036	0.99859	180
Ref 35	*J. Sensors* 5408268 (2019)	0.79	0.9998	100
Ref 36	*IEEE T. Ind. Electron.* **65**, 6487-6496 (2018)	25.7	0.9972	5
Ref 37	*Solid-State Electron.* **139**, 39-47 (2018)	4.65	0.9975	5
Ref 38	*Sensors* **18**, 439 (2018)	30.9	0.9975	0.145
Ref 39	*Sensors* **16**, 1286 (2016)	0.022	0.99915	1000
Ref 40	*Meas. Sci. Technol.* **27**, 124012 (2016)	0.066	0.9967	0.5
Ref 41	*Sensor. Actuat. A-phys.* **228**, 75-81 (2015)	0.02896	0.9979	2500
Ref 42	*Microsyst. Technol.* **21**, 739–747 (2015)	18	0.99876	0.5
Ref 43	*Sensor. Actuat. A-phys.* **216**, 176-189 (2014)	3.68	0.9964	5
Ref 44	*Rev. Sci. Instrum.* **84**, 015004 (2013)	11	0.9695	0.5
Ref 45	*IEEE Sensors* 1-4 (2013)	0.016544	0.9711	0.5
Ref 46	*Microelectron. Eng.* **91**, 167-173 (2012)	0.0279	0.9966	450

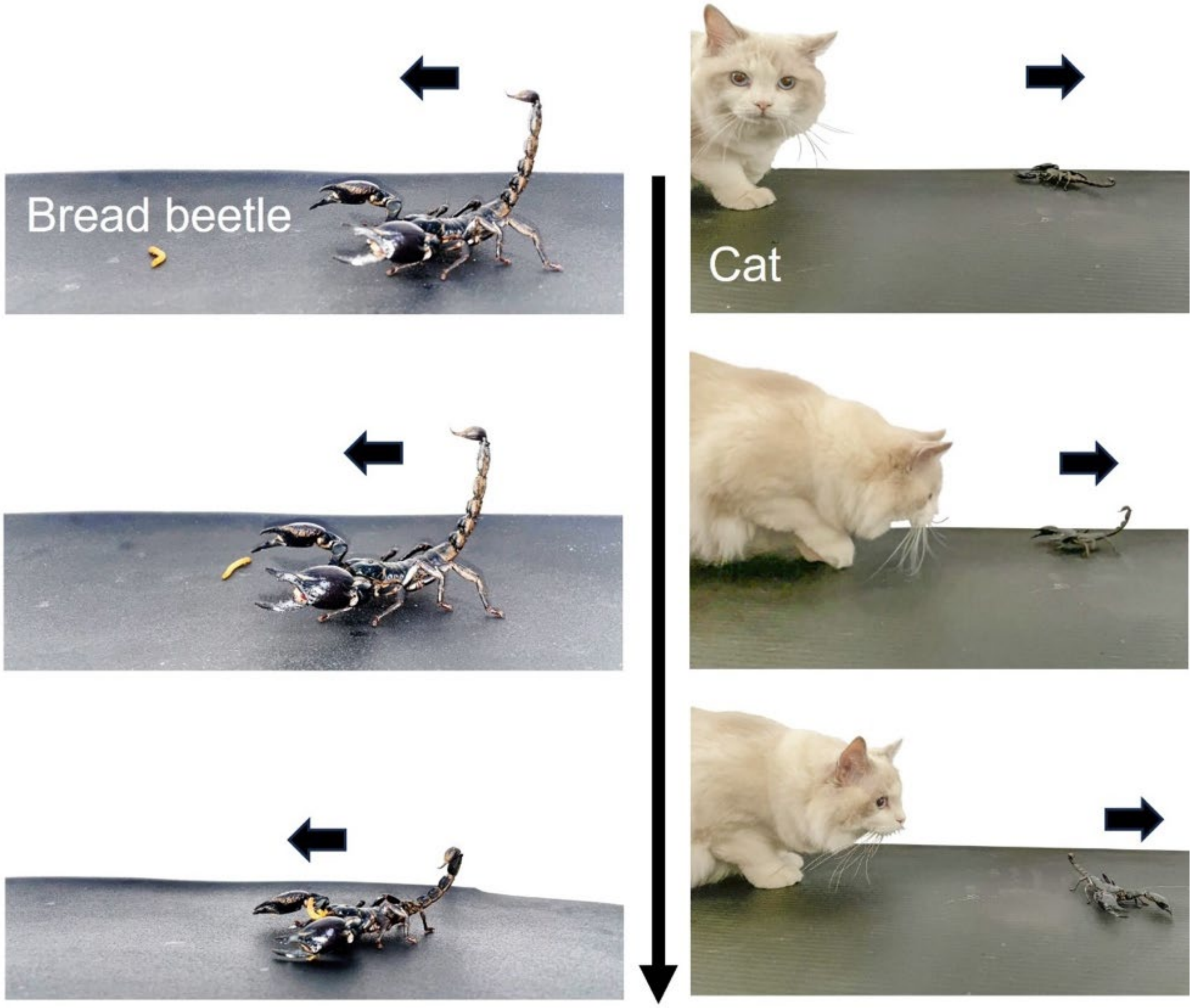

Fig. S42 | Behavioral experiments with the scorpion. a, Predatory behaviors. **b**, Hunting behaviors.

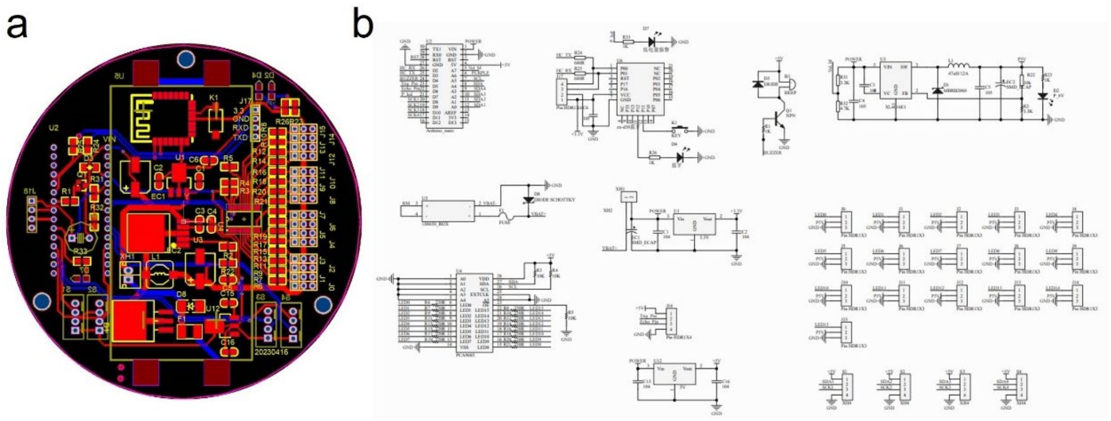

Fig. S43 | The circuit of the hexapod walking platform. a, PCB document. **b**, Schematic diagram document.

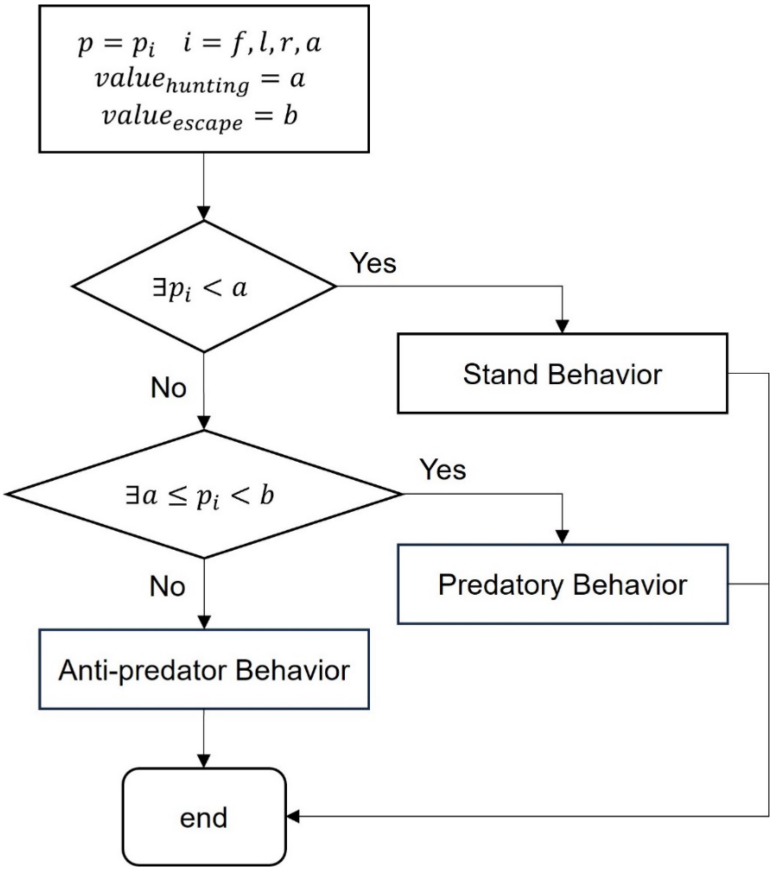

Fig. S44 | Flowchart of a control algorithm for hexapod walking platform. When the robot detects that the pressure p is less than the hunting value v_h, it indicates that there are no prey or natural enemies nearby, and it executes the standing program; When the detection pressure p is between the hunting value v_h and the escape value v_e, it indicates the presence of prey nearby rather than predators, and the hunting program is executed; When the detection pressure p exceeds the escape value v_e, it indicates the presence of natural enemies nearby, and the robot executes the escape program.

Movie S1 | Movie S1 directly shows trichobothria's hypersensitivity perception of weak airflow. We use a high-speed camera to observe the weak air flow response of the trichobothria on the fresh scorpion's pedipalp. As the hands are slightly agitated, the weak airflow caused can be sensitively sensed by the trichobothria.
